# Effect of water and nitrogen coupling regulation on the growth, physiology, yield, and quality attributes and comprehensive evaluation of wolfberry (*Lycium barbarum* L.)

**DOI:** 10.3389/fpls.2023.1130109

**Published:** 2023-06-21

**Authors:** Zhenghu Ma, Juan Yin, Yingpan Yang, Fubin Sun, Zhen Yang

**Affiliations:** ^1^ School of Civil and Water Engineering, Ningxia University, Yingchuan, China; ^2^ Ministry of Education Engineering Research Center for Modern Agricultural Water Resources Efficient Utilization in Dry Areas, Ningxia University, Yingchuan, China; ^3^ Ningxia Water-saving Irrigation and Water Resources Control Engineering Technology Research Center, Ningxia University, Yingchuan, China

**Keywords:** water-nitrogen coupling, photosynthetic daily dynamics, TOPSIS model, composite scoring method, multi-objective optimization, wolfberry

## Abstract

The characteristics of the growing environment (arid and semi-arid regions with abundant light), wastage of water, types of fertilizers used, quality of the plants, and the decline in yield due to the need for large quantities of water and fertilizers are the most significant obstacles to wolfberry cultivation. To cope with the scarcity of water caused by the increase in the area of wolfberry cultivation and to improve the efficiency of the utilization of water and fertilizers, a two-year field experiment was conducted in a typical area of the central dry zone of Ningxia in 2021 and 2022. The effects of different water and nitrogen coupling on the physiology, growth, quality, and yield of wolfberry were investigated, and a water and nitrogen management model with better indicators was constructed based on the TOPSIS model and a comprehensive scoring method. In the experiment, three irrigation quotas of 2,160, 2,565, and 2,970 m^3^ ha^–1^ (I_1_, I_2_, and I_3_) and three N applications of 165, 225, and 285 kg ha^–1^ (N_1_, N_2_, and N_3_) were established; the local conventional management served as the control (CK). The results showed that the growth index of wolfberry was most significantly affected by irrigation, followed by the water and nitrogen interaction effect, and the nitrogen application had the least effect. The growth and development of wolfberry plants mainly takes place during the fruit ripening and flowering periods, and growth almost stops after entering the fruit ripening period. The chlorophyll (SPAD) values were affected by irrigation and nitrogen application to a significant level, except for during the spring tip period, but the effect of water and nitrogen interaction was not significant. The SPAD values of N2 treatment were better under different irrigation. The daily photosynthetic activity of wolfberry leaves peaked between 10:00 am and noon. The daily photosynthetic dynamics of wolfberry were affected by irrigation and nitrogen application to a significant level during the fruit ripening period, and the transpiration rate and leaf water use efficiency were affected by water and nitrogen interaction to a significant level during 8:00 am and noon, while the effect was not significant during the spring tip period. The yield, dry-to-fresh ratio, and 100 grain weight of wolfberry were significantly affected by the irrigation, nitrogen application, and their interaction effects. Specifically, the two-year yield with I_2_N_2_ treatment increased by 7.48% and 3.73%, respectively, compared to CK. The quality indices were significantly affected by irrigation and nitrogen application, except for the total sugars; other indexes were also significantly affected by water and nitrogen interaction effects. The evaluation of the TOPSIS model showed that the I_3_N_1_ treatment yielded the best quality of wolfberry, and the results of the integrated scoring method based on the growth, physiology, yield, and quality indicators and water-saving objectives showed that the I_2_N_2_ (2,565 m3 ha^-1^, 225 kg ha^-1^) treatment was the optimal water and nitrogen management mode for drip-irrigated wolfberry. Our findings provide a scientific basis for the optimal irrigation and management of fertilization of wolfberry in arid regions.

## 1 Introduction

Wolfberry (*Lycium barbarum* L.) is a perennial deciduous shrub in the genus *Lycium* and family Solanaceae (Duan et al., 2022). Its fruits are rich in polysaccharides, total sugars, and other nutrients. It is a medicinal plant with significant beneficial effects on the kidney and liver, as well as enhancing immunity, alleviate fever, and moistening the lungs (Amagase and Farnsworth, 2011; Mao et al., 2011).

The only *Lycium* genus included in the 2010 edition of the Chinese Pharmacopoeia was Ningxia wolfberry. At the end of 2021, 30,000 ha of land was in use for cultivating this wolfberry in Ningxia Province. The cultivated area has increased over the years, and wolfberry has become a specialty crop that contributes to regional economic growth. Wolfberry planting requires a large quantity of water and fertilizers for high yield and income. Thus, wolfberry cultivation has several challenges, including the low availability of water and fertilizers, high-cost, and poor yield and quality. Additionally, whether the excessive use of water and fertilizers contribute to environmental degradation needs to be determined (Liang et al., 2014; Li et al., 2019a).

Water and nitrogen are the most important factors required for crop growth, particularly considering that adding large quantities of nitrogen during crop growth enhances food production and quality (Lebauer and Treseder, 2008; Canfield et al., 2010; Fan et al., 2017). The distribution of sources of water across the globe does not match the distribution of arable land resources, and more than half of agricultural areas are constrained by water availability to varying degrees (Geng et al., 2016). Ningxia is located inland in northwest China. It suffers from extreme water scarcity and the whole region except for the Yellow River Irrigation Area is a fragile ecological environment. Hence, the expansion of wolfberry cultivation in this region will further intensify the water-land conflict. Since the 1980s, the quantity of N fertilizer used in China has increased considerably, and the input-to-output ratio has decreased sharply. The average utilization efficiency of the three fertilizers with high contents of N, P, and K is about 27.2%, 11.1%, and 31.1%, respectively, and the wide northwest area with poor soils has lower utilization efficiency than the national average (Zhang et al., 2008; Wu et al., 2019). Therefore, optimizing water and nitrogen usage and improving water and nitrogen utilization efficiency are essential for improving food security and developing sustainable agricultural techniques (Mahajan et al., 2012; Sun et al., 2016).

Some studies have shown a prominent interaction between water and nitrogen (Cabrera et al., 2007). Proper water conditions promote plant root development, thus enhancing nitrogen uptake efficiency and conversion rate (Huang et al., 2016). In contrast, an inadequate water supply inhibits nitrogen fertilizer and crop growth, and too much will reduce the efficiency of water and fertilizer use, affecting crop yields, and cause a large amount of soil nitrogen loss or leaching into the deep soil layer to cause soil and groundwater pollution (Badr et al., 2010; Tang et al., 2010; Man et al., 2014). Water and fertilizer coupling technology has a synergistic effect on the growth, yield, and quality of crops, and promotes water and fertilizer conservation and green agricultural development (Sasada et al., 2011; Ma et al., 2020). Water and fertilizer coupling technology is widely used in wheat, maize, rice, and facility vegetable cultivation. Although it can effectively save water and fertilizer, its application is mainly focused on annual crops (Chen et al., 2021; Ma et al., 2021; Deng et al., 2022; Wang et al., 2022; Zhao et al., 2022). Few studies have investigated perennial crops such as wolfberry, and most studies have examined the interactive effects between irrigation and various N, P, and K fertilizers. The identification of the reciprocal effects of irrigation and a specific fertilizer and their mechanism of action is challenging (Liu et al., 2020; Deng et al., 2021; Zhu et al., 2022). Thus, the effect of saving water and fertilizer is not obvious after applying this technology. Therefore, the application of water and fertilizer coupling technology for cultivating perennial crops and optimizing irrigation systems needs further investigation.

The limitations of the distribution area and the specificity of the growth habit have discouraged the development of irrigation systems and related studies on wolfberry. Thus, the effects of water, nitrogen, and their interactions on wolfberry plants need to be determined to use water and nitrogen efficiently in wolfberry cultivation. To address some of these issues, we conducted this study with the following objectives: (1) to determine the effects of irrigation, nitrogen application, and their interaction on the growth, physiology, yield, and quality of wolfberry by conducting a two-year field experiment; (2) to develop an evaluation system based on the physiology, growth, and quality of wolfberry by applying the TOPSIS model and the integrated scoring method; (3) to determine the optimal irrigation quota and content of nitrogen required for the drip irrigation of wolfberry with high efficiency of water and nitrogen utilization, healthy growth of the plants, excellent yield, and high quality of the crop. This study might provide a theoretical foundation and support for developing an intelligent drip irrigation system for wolfberry production.

## 2 Materials and methods

### 2.1 Study area

**Figure 1:**
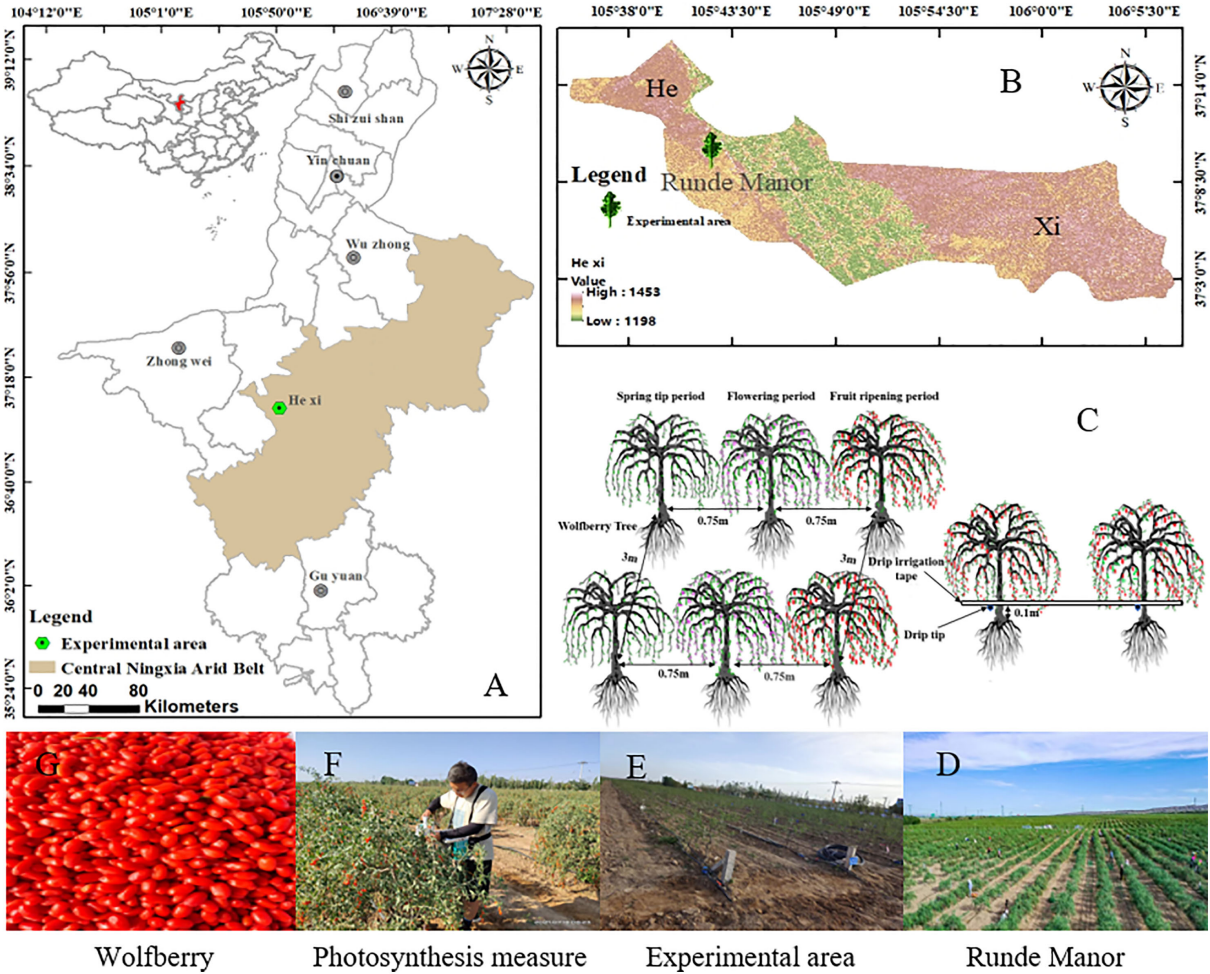
Ningxia, China **(A)**, Study Area Location - Hexi Town **(B)**, Experimental design **(C)**, Rundle Manor **(D)**, Experimental area **(E)**, Photosynthesis measure **(F)** and wolfberry **(G)**.

**Figure 2:**
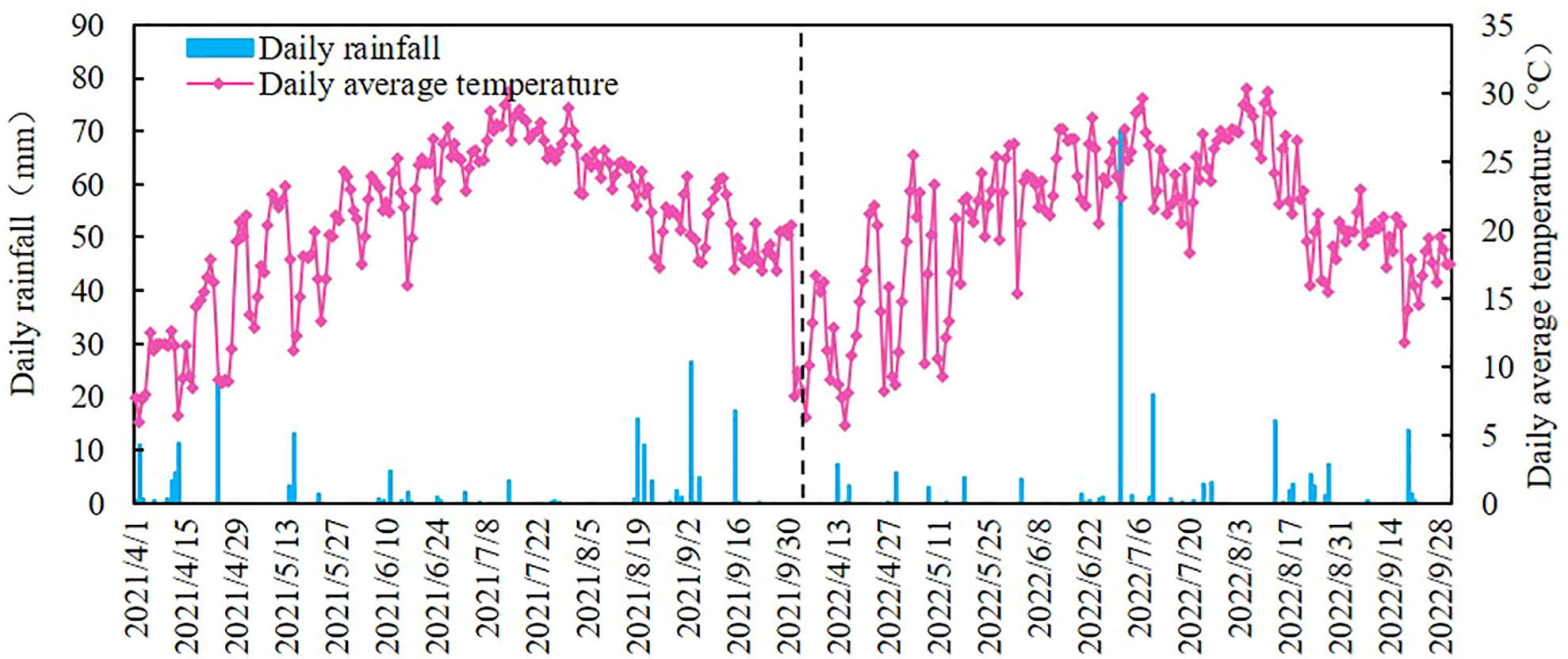
The daily rainfall and daily average temperature during the reproductive period of wolfberry in 2021 and 2022.

The study was conducted in Ningxia Concentric County, China (105°42’23.05” E, 37°10’36.98” N, 1,228 m). It is a typical arid belt region (**Figure 1**). The study was conducted from April 2021 to October 2022. The area has a temperate continental semi-arid climate, with an average rainfall of 270 mm; approximately 70% of the annual rainfall occurs between July and September. The drought indicator is approximately 8.84, and thus, supply and demand have a great disparity. The average annual temperature has been 8.6°C for many years, with a large variation in diurnal temperature and an average of approximately 3,024 h of sunlight. The porosity of the soil is 44%, and the field water holding capacity ranges from 13.6% to 15.4%. The physicochemical properties of the soil are listed in **Table 1**. The groundwater of this region is buried between 20 and 25 m deep and precipitation is minimal and concentrated, making the groundwater and precipitation difficult to use and the only reliable source of water for irrigation is the Yellow River. The precipitation and average daily temperature throughout the fertility period in the two years of the study are shown in **Figure 2**. The experimental varieties of wolfberry were 8a and 9a Sheng Ningqi No. 7. The plants were grown 0.75 m apart, and a line distance of 3 m was maintained. Wolfberry trees have a trunk diameter of 30–45 mm and a height of 80–110 cm at the start of their reproductive period. They have a four-stage critical reproductive period: the spring tip period (stage 1: late April to mid-May), the flowering period (stage 2: late May to mid-June), the fruit ripening period (stage 3: late June to mid-August), and the defoliation period (stage 4: late June to mid-August) (stage 5: late August to early September).

**Table 1:** Physicochemical properties of the soil in the study area.

Year	Depth	pH	EC	Total N	Total P	Total K	Organic material	Ammonium N	Nitrate N
	cm		μs·cm^-1^	g·kg^-1^				mg·kg^-1^	
2021	0-20	8.00	1615	0.46	0.43	16.19	7.58	12.67	14.69
	20-40	8.20	1091	0.42	0.42	17.15	6.73	4.51	4.73
2022	0-20	8.06	1524	0.45	0.40	17.00	7.21	12.24	14.17
	20-40	8.12	963	0.41	0.41	17.00	6.28	4.42	4.82

### 2.2 Experimental design

The fully randomized experimental design consisted of two factors: irrigation and nitrogen fertilization. Three irrigation amounts, i.e., low water I_1_ (2,160 m^3^ ha^-1^), medium water I_2_ (2,565 m^3^ ha^-1^), and high water I_3_ (2,970 m^3^ ha^-1^), and three N application rates, i.e., low N_1_ (165 kg ha^-1^), medium N_2_ (225 kg ha^-1^), and high N_3_ (285 kg ha^-1^), were used in the experiment following the drip irrigation planting technical protocols (Ningxia Water Resources Department and Ningxia Institute of Water Resources Science, 2017; Zhang, 2021). The experimental design levels are shown in **Table 2**. The local conventional management standard was used as the control (CK) with the following irrigation quotas, N, P, and K pure nutrients: 2,970 m^3^ ha^-1^, 330 kg ha^-1^, 90 kg ha^-1^, and 150 kg ha^-1^, respectively. Each plot had 10 trees with more than 3 m of separation between each plot of wolfberry trees to prevent mutual influence, and the plot area was 22.5 m^2^. The experiment included nine treatments and one control treatment, with three replications for each treatment, and 30 test plots. A 6 m buffer zone surrounding each test plot was maintained, and the total test area was 675 m^2^. Urea (46% N), calcium superphosphate (12% P_2_O_5_), and potassium sulfate (50% K_2_O) were used as the test fertilizers. The phosphorus (P_2_O_5_) and potassium (K_2_O) fertilizers were applied quantitatively, based on the relevant results, and set at 66 and 113 kg ha^-1^, respectively (Deng et al., 2021; Zhang, 2021). We performed above-ground, off-frame drip irrigation, and the drip pipe was laid parallel to each row. Irrigation and nitrogen application were synchronized as follows: 20% and 15% (once) during the spring tip period, 30% and 25% (twice) during the flowering period, 40% and 50% (four times) during the fruit ripening period, and 10% (once) during both defoliation periods. Except for irrigation and fertilization, all other field management practices and levels were identical to those of the surrounding area.

**Table 2:** The water and nitrogen management program of wolfberry.

Fertility stages	2021							2022						
	Date	Irrigation amount m^2^ ha^-1^			Nitrogen amount kg ha-1			Date	Irrigation amount m^2^ ha^-1^			Nitrogen amount kg ha^-1^		
		I_1_	I_2_	I_3_	N_1_	N_2_	N_3_		I_1_	I_2_	I_3_	N_1_	N_2_	N_3_
Spring tip period	04-30	432	513.0	594	24.75	33.75	42.75	04-28	432	513.0	594	24.75	33.75	42.75
Flowering period	05-23	216	256.5	297	12.38	16.88	21.38	05-23	216	256.5	297	12.38	16.88	21.38
	06-18	432	513.0	594	28.88	39.38	49.88	06-15	432	513.0	594	28.88	39.38	49.88
Fruit ripening period	07-05	216	256.5	297	20.63	28.13	35.63	07-02	216	256.5	297	20.63	28.13	35.63
	07-15	216	256.5	297	20.63	28.13	35.63	07-15	216	256.5	297	20.63	28.13	35.63
	07-25	216	256.5	297	20.63	28.13	35.63	07-28	216	256.5	297	20.63	28.13	35.63
	08-05	216	256.5	297	20.63	28.13	35.63	08-08	216	256.5	297	20.63	28.13	35.63
Defoliation period	08-25	216	256.5	297	16.50	22.50	28.50	08-28	216	256.5	297	16.50	22.50	28.50
Total		2160	2565	2970	165	225	285		2160	2565	2970	165	225	285

### 2.3 Observation indicators and methods

#### 2.3.1 Growth indicators

After performing the initial irrigation, three wolfberry trees with comparable growth were selected from each plot. In the upper portion of each subdivision, three new unshaded branches were delineated and measured, and the east-west and south-north crown widths were marked.

The height, length, and diameter of the selected calibrated branches were measured with an accuracy of 0.1 mm using steel measuring tape and Vernier calipers. The east-west and south-north crown widths of each tree were measured with an accuracy of 0.1 mm using steel measuring tape. The first monitoring of each index was carried out on the 10th day after irrigation, and then measured once every 10 days.

#### 2.3.2 Physiological indicators

Three leaves were selected from the demarcated branches, and the leaf SPAD value was determined using a portable (measured every 10 days), hand-held chlorophyll instrument (SPAD-502PLUS, Japan). On a clear and non-windy day, three healthy and uniformly illuminated leaves were selected and monitored every 2 h from 08:00 am to 6:00 pm using a portable photosynthetic measurement system (LI-6800, USA). The monitoring indicators included the net photosynthetic rate (*Pn*), the transpiration rate (*Tr*), and stomatal conductance (*Gs*). The leaf water use efficiency (*WUE*) was calculated using the formula *WUE* = *Pn*/*Gs*.

#### 2.3.3 Yield and yield composition indicators

At the ripening stage, ripe fruits were collected in batches. The fresh fruit yield and weight of 100 fresh wolfberries were determined using an electronic scale (0.01 g). After drying the fruits, their yield was calculated. To determine the number of grains, the 100-grain weight of the dried fruits was weighed, and 50 g of the dried fruit was randomly weighed in each plot. Finally, the dry-fresh ratio was calculated based on the yield of dried fruits and fresh fruits.

#### 2.3.4 Quality measurement

For each treatment, 500 g of dried fruits were randomly selected for quality determination, and the determination indices included the polysaccharide content, total sugar content, crude fat content, protein content, and betaine content. Polysaccharides were determined by spectrophotometry at 490 nm absorbance, and total sugars were determined using the titration method (General Administration of Quality Supervision, Inspection and Quarantine of the People’s Republic of China and China National Standardization Administration Committee, 2014). The Kjeldahl method was used to determine the protein content (National Health and Family Planning Commission of the People’s Republic of China, 2016a). Betaine was determined using the colorimetric method with a detection limit of 0.04% (Ministry of Agriculture and Rural Affairs of the People’s Republic of China, 2009). The Soxhlet extraction method was used to determine the crude fat content (National Health and Family Planning Commission of the People’s Republic of China, 2016b).

### 2.4 Multi-objective decision-making and comprehensive evaluation

#### 2.4.1 Comprehensive scoring method

Each evaluation indicator was scored according to the evaluation criteria of different indicators. Then, the weighted sum was used to obtain the total score.

Suppose the number of evaluation objects and evaluation indicators are *a* and *b*, respectively, and $X_{ij}$ represents the *j* ^th^ indicator of the *i* ^th^ treatment. Then, the normalized indicator $Y_{ij}$ can be obtained by eliminating the dimensionality of the measured values of each indicator.


(1) $$Y_{ij}=\frac{X_{ij}-min\left(X_{ij}\right)}{max\left(X_{ij}\right)-min\left(X_{ij}\right)}\left(\begin{array}{c} i=1,2,\cdots \text{,a}\\ j=1,2,\cdots \text{,b} \end{array}\right)$$

The indicator weight was determined by the coefficient of variation method of objective weighting. Because the dimensions of the indicators were different, comparing their variability directly was not possible. Thus, the coefficient of variability of the indicators was used to compare their variability (Sun et al., 2022).


(2) $${\text{V}}_{\text{j}}=\frac{{\text{δ}}_{\text{j}}}{{\text{χ}}_{\text{j}}}$$

(3) $${\text{W}}_{\text{j}}=\frac{{\text{V}}_{\text{j}}}{\sum_{1}^{n}{\text{V}}_{\text{j}}}$$

Here, *V_j_* indicates the coefficient of variation of indicator *j*; *δ_j_* indicates the standard deviation of indicator *j*; *χ_j_* indicates the mean of indicator *j*; *W_j_* indicates the weight of indicator *j*.

The comprehensive score of the treatment $C_{si}$ was calculated using equation (4).


(4) $${\text{C}}_{\text{si}}=\sum_{\text{j}=1}^{\text{b}}{\text{W}}_{\text{j}}{\text{Y}}_{\text{ij}}$$

#### 2.4.2 TOPSIS method

(1) The matrix was constructed based on the original evaluation parameters (Liu et al., 2021a). Suppose there are m evaluation objects and n evaluation indicators. The original data can be expressed as the matrix X = ( ${\text{X}}_{ij}$ ) m×n; $X_{ij}$ represents the original data of the *j* ^th^ indicator of the *i* ^th^ treatment. The metrics was normalized as follows:


(5) $$Z_{ij}=\frac{X_{ij}}{\sqrt{\sum_{j=1}^{n}{\text{X}}_{ij}^{2}}}$$

Here, i = (1, 2, …, m) and j = (1, 2, …, n)

The normalization matrix can be expressed as Z = ( $Z_{ij}$ ) *m×n*. The optimal and inferior vectors composed of the maximum and minimum values of each column were determined using equations (6) and (7), respectively.


(6) $$Z^{+}=(Z_{max1}\text{, }Z_{max2}\text{, }\cdots \text{, }Z_{maxn})$$

(7) $$Z^{-}=(Z_{min1}\text{, }Z_{min2}\text{, }\cdots \text{, }Z_{minn})$$

The Euclidean distances (D_i_^+^ and D_i_^–^) were determined using equations (8) and (9).


(8) $$D_{i}^{+}=\sqrt{\sum_{\text{j}=1}^{\text{n}}{{\text{(Z}}_{\text{max j}}{\text{ - Z}}_{\text{ij}})}^{2}}$$

(9) $$D_{i}^{-}=\sqrt{\sum_{\text{j}=1}^{\text{n}}{{\text{(Z}}_{\text{max j}}{\text{ - Z}}_{\text{ij}})}^{2}}$$

The fit of the *i* ^th^ treatment to the optimal solution $C_{\text{i}}$ was determined using equation (10).


(10) $$C_{i}=\frac{D_{i}^{-}}{\left(D_{i}^{+}+D_{i}^{-}\;\right)}$$

### 2.5 Data processing and analysis

All data were collated and model calculations were performed in Microsoft Excel 2018. The analysis of variance (ANOVA) was performed on the SPSS 26.0 software to determine the effect of water, nitrogen, and their interaction, and its interaction effects with significance (P< 0.05). The graphs were plotted using Microsoft Excel, GraphPad Prism 8.0, and Origin 21.0.

## 3 Results and analysis

### 3.1 Growth indicator

#### 3.1.1 Cumulative growth

**Figure 3:**
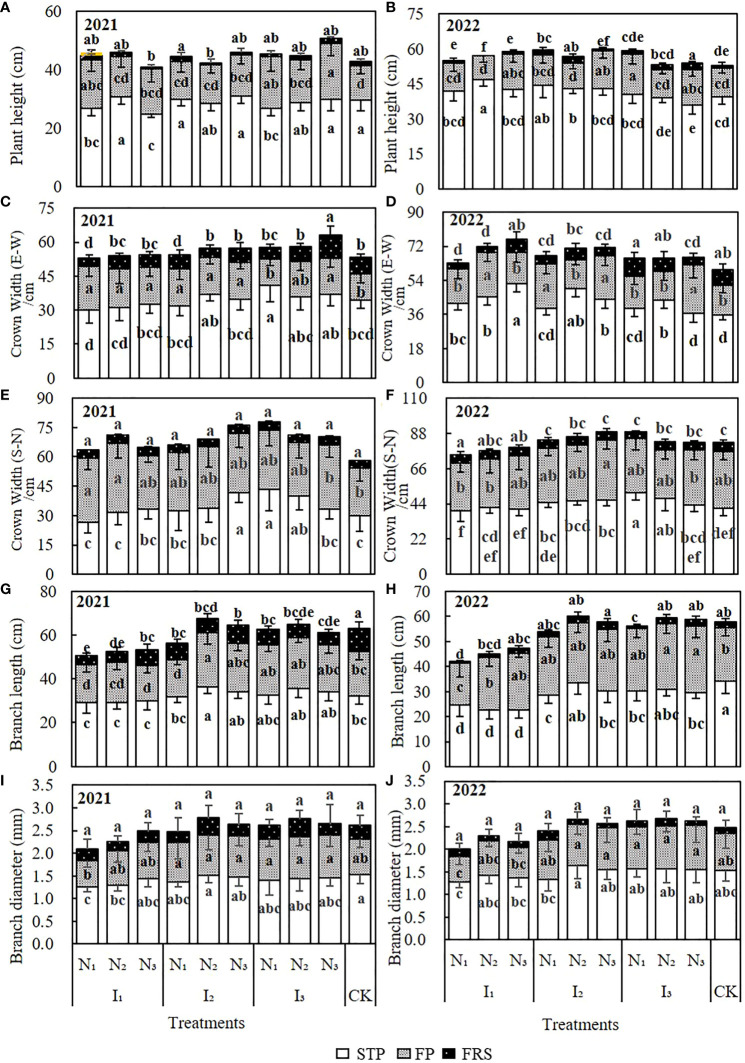
The effect of water and nitrogen coupling on the cumulative growth of wolfberry plant height **(A, B)**, crown width (E–W) **(C, D)**, crown width (S–N) **(E, F)**, branch length **(G, H)**, branch diameter **(I, J)** in the fertility periods of 2021 and 2022. The different lowercase letters indicate significant differences, as determined by Duncan’s multiple range test (p < 0.05). CK, control; STP, spring tip period; FP, flowering period; FRP, fruit ripening period.

The cumulative increase in the growth indices of wolfberry plants in 2021 and 2022 is shown in **Figure 3**. The growth and development of wolfberry plant height, crown width (E-W), crown width (S-N), branch length, and branch diameter growth indices mainly occurred during the spring tip and flowering periods.

The two-year cumulative plant height was 41–50.78 cm and 52.89–59.89 cm, respectively (**Figures 3A, B**). The maximum cumulative height was recorded during the spring tip period of 2022; the maximum and minimum heights were observed in the I_3_N_3_ and I_1_N_3_ treatments in 2021, and in the I_2_N_3_ and CK treatments in 2022, respectively. When water and nitrogen were coupled, the plant height differed between treatments at different growth periods. However, the average plant height did not change significantly with irrigation and nitrogen gradient over the two years.

The cumulative crown width (E-W) growth in 2021 and 2022 was 53–63.11 cm and 59.56–75.77 cm, respectively (**Figures 3C, D**). The highest growth occurred in the I_3_N_3_, and I_1_N_3_ treatments, and the poorest growth occurred in the I_1_N_1_ and CK treatments. The cumulative growth of the crown width (E-W) increased with the increase in the irrigation quota and nitrogen application in 2021, but the opposite trend was observed in 2022. There were some differences in the cumulative growth of the crown width (E-W) in different fertility periods with water and nitrogen treatments, but the differences were not significant for the whole fertility period.

The cumulative growth of the crown width (S-N) in 2021 and 2022 was 58.22–77.78 cm and 74.78–89.11 cm, respectively (**Figures 3E, F**). The highest growth occurred in the I_1_N_3_ and I_2_N_3_ treatments in 2021 and 2022, respectively. The crown width (S-N) growth increased significantly with an increase in nitrogen application under the I_1_ and I_2_ irrigation quotas, while under the I_3_ irrigation quota, the increase in nitrogen application decreased the crown width (S-N) growth.

The cumulative growth of the branches was 50.67–67.44 cm in 2021 and 41.91–60.22 cm in 2022 (**Figures 3G, H**). The maximum and minimum branch lengths were recorded in the I_2_N_2_ and I_1_N_1_ treatments. Under I_1_ irrigation, the branch length increased gradually with nitrogen application, whereas, under I_2_ and I3 irrigation, it increased initially and then decreased slightly. The interaction between water and nitrogen significantly affected the branch length.

The branch diameter changes in 2021 and 2022 were generally consistent (**Figures 3I, J**), with cumulative growth ranging from 2.08–2.77 mm and 2–2.68 mm, respectively. The maximum growth in branch diameter was observed in the spring and flowering seasons. Under I_1_ irrigation, the branch diameter increased gradually with an increase in N application. Under I_2_ and I_3_ irrigation, the diameter first increased and then slightly decreased. However, in some treatments, the diameter differed significantly (P< 0.05) during the spring tip and flowering periods, and they were significantly influenced by water and nitrogen coupling.

#### 3.1.2 Analysis of variance for each growth indicator

The results of the ANOVA are shown in **Table 3**. Except for the fruit ripening stage in 2021, the effects of irrigation on plant height, nitrogen application during the flowering period, and water and nitrogen coupling during the spring tip period were significant. The crown width was significantly influenced by the interaction between irrigation water and nitrogen. The crown width (E-W) experienced a greater influence at all reproductive stages. The crown width (S-N) was only significantly greater during the spring tip period. Irrigation significantly affected the branch length at all growth stages. Flowering and fruit ripening were significantly affected by water, nitrogen, and the coupling of water and nitrogen. The diameter of the branches was significantly affected only by irrigation. Comprehensive analysis showed that irrigation had the greatest effect on the cumulative growth of the indicators, followed by the interaction between water and nitrogen, and then, by nitrogen application.

**Table 3:** The results of the analysis of variance for each growth index of wolfberry with water and nitrogen coupling.

Indices	Treatment	Spring tip period		Flowering period		Fruit ripening period	
		2021	2022	2021	2022	2021	2022
Plant height	I	**	**	**	*	ns	**
	N	ns	*	*	**	ns	ns
	I×N	**	*	ns	ns	ns	**
Crown width (E-W)	I	**	**	*	ns	**	**
	N	ns	**	ns	*	**	ns
	I×N	*	**	ns	**	**	**
Crown width (S-N)	I	**	**	ns	ns	ns	ns
	N	ns	ns	ns	ns	ns	ns
	I×N	**	*	ns	ns	ns	ns
Branch length	I	**	**	**	**	**	**
	N	ns	ns	**	*	ns	**
	I×N	ns	ns	**	ns	*	ns
Branch diameter	I	ns	**	**	**	ns	ns
	N	ns	ns	ns	ns	ns	ns
	I×N	ns	ns	ns	ns	ns	ns

**indicates a highly significant effect (P< 0.01), *indicates a significant effect (P< 0.05), and ns indicates no significant effect (P > 0.05); I, irrigation quota; N, the amount of nitrogen applied; E-W, East-West; S-N, South-North.

### 3.2 Physiological indicators

#### 3.2.1 Leaf SPAD value

**Figure 4:**
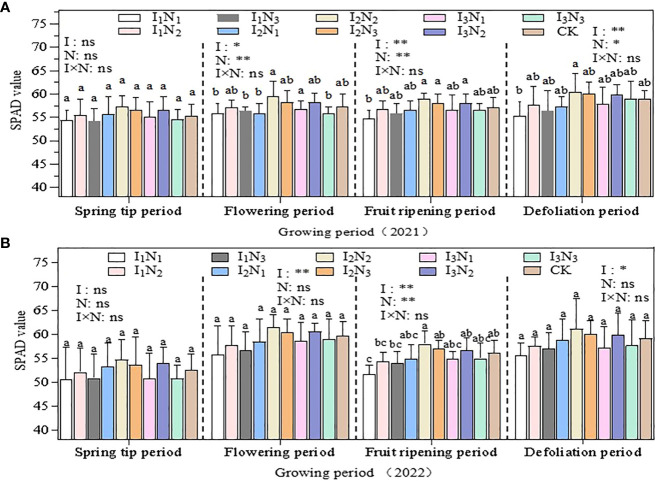
The effect of water and nitrogen coupling on the SPAD value of wolfberry leaves in 2021 **(A)** and 2022 **(B)**. Note that different lowercase letters indicate significant differences between different water and nitrogen treatments. ** indicates a highly significant effect (p < 0.01); * indicates a significant effect (p <0.05); and ns indicates no significant effect (p > 0.05). I, irrigation quota; N, amount of nitrogen applied.

The pattern of variation in the leaf SPAD values was similar between 2021 and 2022 (**Figures 4A, B**), with a gradual increase from the spring tip period to the flowering period, a slight decrease during the fruit ripening period, and a gradual increase during the defoliation period, which was more pronounced in 2022. There were significant differences (P< 0.05) among other treatments, such as among I_2_N_2_, I_2_N_3_, and I_3_N_2_ during the flowering, fruit ripening, and defoliation periods in 2021, and only between I_1_N_1_ treatment and I_2_N_2_, I_2_N_3_, and I_3_N_2_ with medium-to-high water fertilization during the fruit ripening period in 2022 (P< 0.05). The irrigation quotas andnitrogen application in the 2 years of the study had significant (p < 0.05) effects at the flowering, fruit ripening, and defoliation periods, but none of the effects of water and nitrogen interactionreached significant levels. The SPAD maximum values in both years were recorded in the I_2_N_2_ treatment and, under the same irrigation level, the SPAD values first increased and then decreased with the increase in nitrogen application, and the overall SPAD values were relatively higher in the N_2_ nitrogen treatment.

#### 3.2.2 Net photosynthetic rate

**Figure 5:**
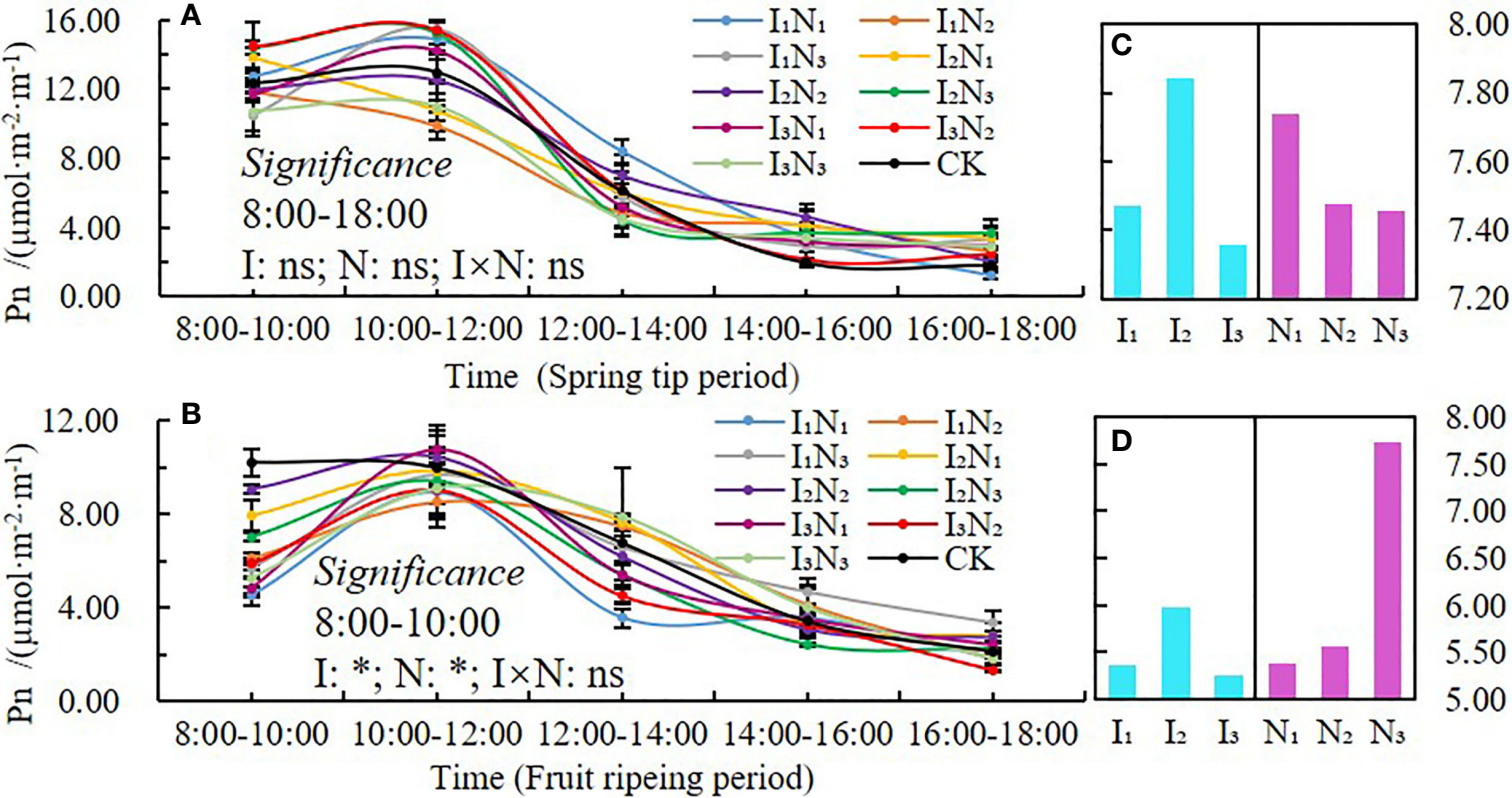
Daily dynamics of and changes in the net photosynthetic rate under conditions of water and nitrogen coupling, Pn **(A, B)**, Changes with irrigation and N application **(C, D)**. Note that photosynthetic day dynamics at different fertility stages were expressed as average values in 2021 and 2022, the same applies in **Figures 6**–**8**. * indicates a significant effect (p < 0.05); ns indicates no significant effect (p > 0.05).

The daily dynamics of the net photosynthetic rate (*Pn*) in 2021 and 2022 are shown in **Figure 5**. A single-peaked pattern was observed during both fertility periods (**Figures 5A, B**), and the highest peaks occurred between 10:00 am and noon. During the spring tip period, the net photosynthetic rate was higher in the I_1_N_3_ and I_2_N_3_ treatments, and the highest value reached 18.25 µmol·m^-2^;·s^-1^, which occurred in the I_1_N_3_ treatment. The fruit ripening period was relatively short and relatively long under the I_2_N_2_ and I_3_N_1_ treatments, with the highest net photosynthetic rate of 12.63 µmol·m^-2^·s^-1^ (I_1_N_3_ treatment). The net photosynthetic rate was not significantly affected by irrigation quota, nitrogen application, and their interaction during the spring tip period but was significantly (P< 0.05) affected by irrigation quota and nitrogen application between 8:00 am and 10:00 am during the fruit ripening period. The net photosynthetic rate initially increased and then decreased in response to an increase in the irrigation water; however, the effect of nitrogen application varied across growth stages (**Figure 5C**). The net photosynthetic rate decreased with an increase in nitrogen application during the spring tip period but increased during the fruit ripening period (**Figure 5D**).

#### 3.2.3 Transpiration rate

**Figure 6:**
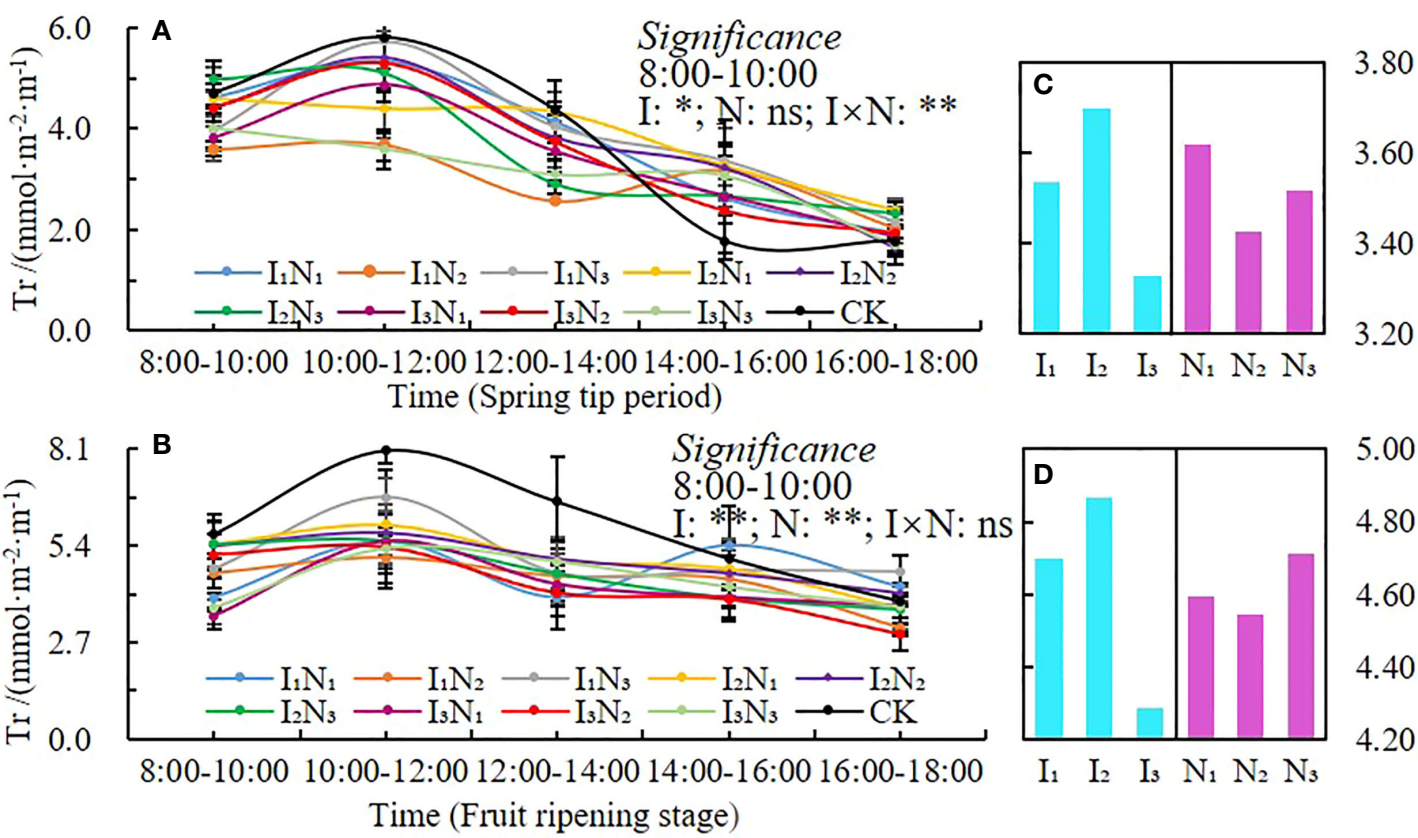
Daily dynamics of and changes in transpiration rate under conditions of water and nitrogen coupling, Tr **(A, B)**, Changes with irrigation and N application **(C, D)**. ** indicates a highly significant effect (p < 0.01); * indicates a significant effect (p < 0.05); and ns indicates no significant effect (p > 0.05).

The daily dynamics of transpiration rate (*Tr*) are shown in **Figures 6A, B**; two peaks were recorded during both fertility periods. The first peak occurred between 10:00 am and noon, which was followed by a decline with a low transpiration rate between noon and 2:00 pm. A second peak occurred between 2:00 pm and 4:00 pm. At peak times, the transpiration rates in the I_1_N_3_, I_2_N_2_, I_3_N_2_, and CK treatments were more than those in the I_1_N_3_, I_2_N_2_, and CK treatments. The transpiration rate between 8:00 am and 10:00 am was affected by the irrigation quota, water, and nitrogen interaction during the spring tip period and by the irrigation quota and nitrogen application during the fruit ripening period; both reached significant levels (P< 0.05). The transpiration rate first increased and then decreased with an increase in irrigation at various fertility stages. Additionally, the rate first increased and then decreased with an increase in nitrogen application (**Figures 6C, D**).

#### 3.2.4 Stomatal conductance

**Figure 7:**
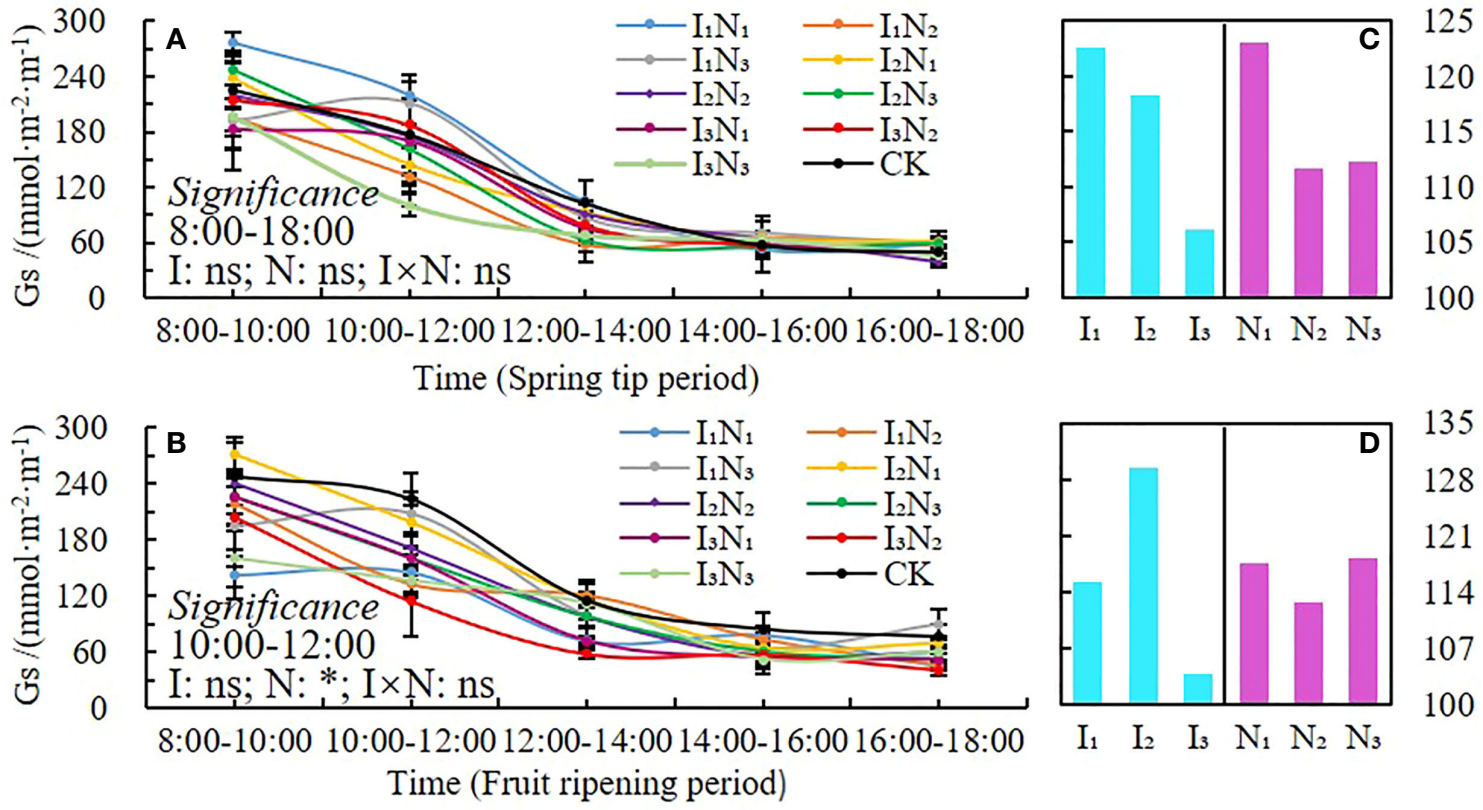
Daily dynamics of and changes in stomatal conductance under conditions of water and nitrogen coupling, Gs **(A, B)**, Changes with irrigation and N application **(C, D)**. * indicates a significant effect (p < 0.05); and ns indicates no significant effect (p > 0.05).

The daily dynamics of stomatal conductance (*Gs*) in 2021 and 2022 are shown in **Figure 7**. A single-peaked pattern for both fertility periods (**Figures 7A, B**) started between 8:00 am and 10:00 am, which peaked between 10:00 am and noon, followed by a sharp decline and a slight increase between 2:00 pm and 4:00 pm. During the peak, the stomatal conductance in the I_1_N_1_ and I_2_N_1_ treatments was relatively high at all reproductive stages. Stomatal conductance was not significantly affected by irrigation quota, nitrogen application, or their interactions during the spring tip period, but it was significantly (P< 0.05) affected by nitrogen application between 10:00 am and noon during the fruit ripening period. During the spring tip period, the stomatal conductance decreased gradually as irrigation water increased (**Figure 7C**). In contrast, during the fruit ripening period, it increased initially and then decreased as irrigation water increased. Additionally, the stomatal conductance initially decreased and then increased as nitrogen application increased (**Figure 7D**).

#### 3.2.5 Leaf water use efficiency

**Figure 8:**
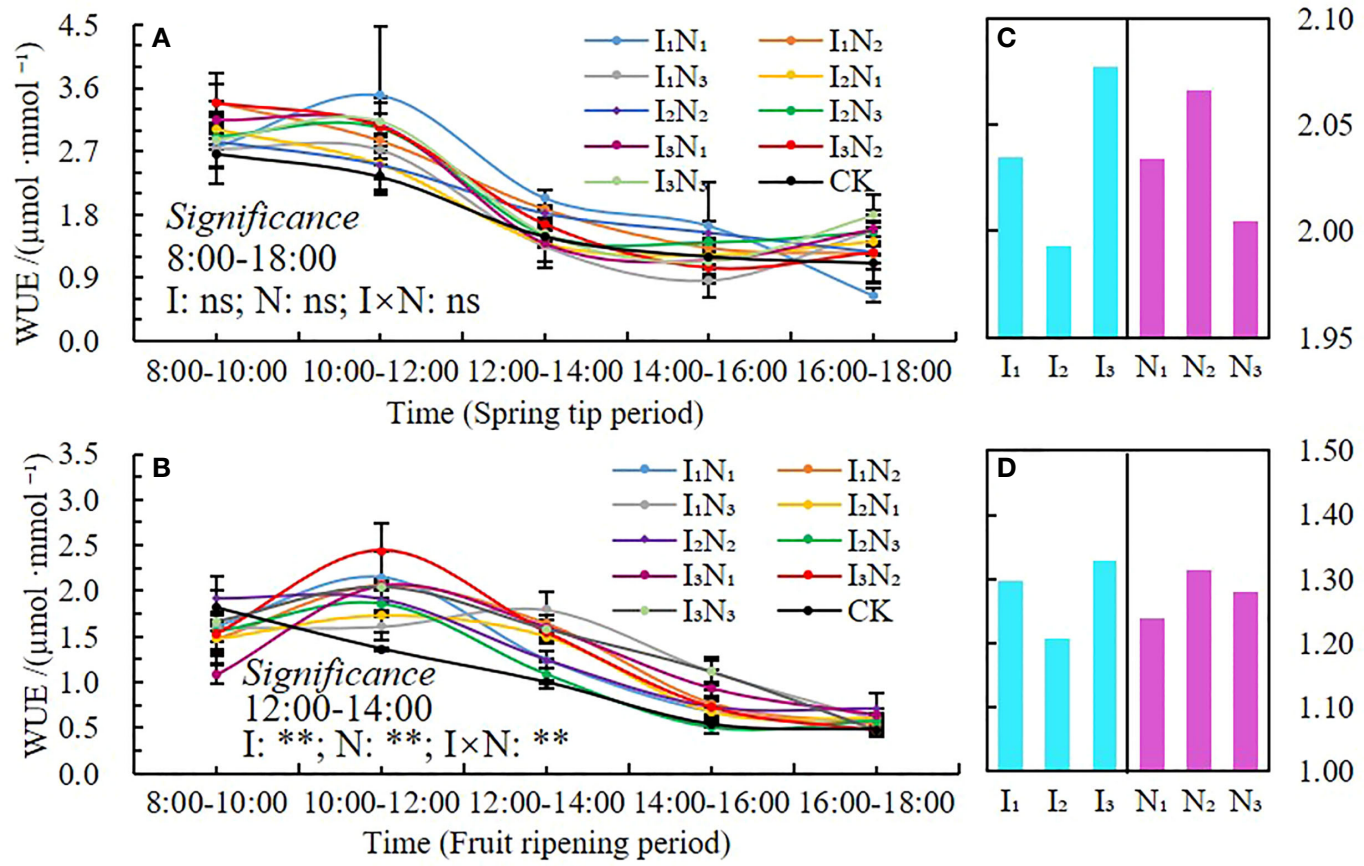
Daily dynamics of and changes in water use efficiency under conditions of water and nitrogen coupling, WUE **(A, B)**, Changes with irrigation and N application **(C, D)**. ** indicates a highly significant effect (p < 0.01); ns indicates no significant effect (p > 0.05).

The daily dynamic characteristics of leaf water use efficiency (*WUE*) in 2021 and 2022 are shown in **Figure 8**. The two growth periods showed a single peak (**Figures 8A, B**), which occurred between 10:00 am and noon, followed by a precipitous decline. The water use efficiency of each treatment was greater in the morning than in the afternoon. The water use efficiency of I_1_N_1_ treatment was the highest (5.27 µmol·mmol^-1^), and it was 49.47% and 56.93% higher than that of CK during the spring tip and fruit ripening periods. Leaf water use efficiency was not significantly affected by irrigation rate, nitrogen application, and their interaction during the spring tip period. However, it was significantly affected by leaf irrigation rate, nitrogen application, and their interaction between noon and 2:00 pm during the fruit ripening period (P< 0.01). The water use efficiency of wolfberry leaves decreased initially, then increased with an increase in irrigation water and decreased with an increase in fertilizer application (**Figures 8C, D**).

### 3.3 Yield and yield composition

The analysis of the yield indicators for wolfberry in 2021 and 2022 is presented in **Table 4**. The yield in both years ranged from 1,953 to 2,320.94 kg ha^-1^ and 1,924.87 to 2,391.73 kg ha^-1^, respectively. The I_2_N_2_ treatment produced the best results, with 7.48% and 3.73% higher yields compared to that in CK and 18.84% and 24.25% higher yields compared to that in the treatment with the lowest yield, respectively. The average yield for both years followed the order I_2_N_2_ > I_3_N_2_ > I_2_N_3_ > CK > I_3_N_3_ > I_3_N_1_ > I_2_N_1_ > I_1_N_3_ > I_1_N_2_ > I_1_N_1_. The dry-to-fresh ratio increased with an increase in irrigation and N application; the CK treatment exhibited the greatest increase. Both high water and high fertilizer and low water and low fertilizer treatments were associated with the highest grain yield.

**Table 4:** The effect of water and nitrogen coupling on yield and yield composition.

Year	Treatment	Fruit yield (kg ha^-1^)	Hundred grains weight (g)	Dry-to-fresh ratio	Number of grains (50g)
2021	I_1_N_1_	1953.00 c	17.40 abc	4.26 b	293.00 abc
	I_1_N_2_	1956.16 c	16.79 c	4.28 ab	295.67 ab
	I_1_N_3_	1984.41 c	18.21 a	4.32 a	279.67 c
	I_2_N_1_	2020.84 c	17.91 ab	4.29 ab	282.00 bc
	I_2_N_2_	2320.94 a	17.24 bc	4.28 ab	288.67abc
	I_2_N_3_	2230.26 ab	17.59 abc	4.26 b	290.67 abc
	I_3_N_1_	2172.74 b	17.39 bc	4.13 d	293.67 abc
	I_3_N_2_	2221.63 ab	17.96 ab	4.20 c	297.33 ab
	I_3_N_3_	2148.89 b	17.25 bc	4.25 bc	297.33 ab
	CK	2159.34 b	17.34 bc	4.32 a	302.33 a
F value of ANOVA	I	**	ns	**	ns
	N	**	ns	**	ns
	I×N	**	**	**	ns
2022	I_1_N_1_	1924.87 f	14.95 c	4.18 d	321.33 ab
	I_1_N_2_	1983.14 ef	15.01 c	4.23 bcd	322.00 ab
	I_1_N_3_	2035.97 def	15.34 bc	4.29 bcd	310.33 b
	I_2_N_1_	2125.74 cde	15.34 bc	4.27 bcd	311.67 b
	I_2_N_2_	2391.73 a	16.54 a	4.20 cd	315.33 6b
	I_2_N_3_	2307.75 ab	16.34 ab	4.19 cd	308.33 b
	I_3_N_1_	2192.29 bcd	15.23 c	4.34 abc	319.33 ab
	I_3_N_2_	2333.85 ab	15.66 abc	4.36 ab	323.33 ab
	I_3_N_3_	2227.90 abc	15.80 abc	4.38 ab	319.33 ab
	CK	2305.82 ab	14.77 c	4.48 a	338.33 a
F value of ANOVA	I	**	**	**	ns
	N	**	*	ns	ns
	I×N	ns	ns	ns	ns

Different lowercase letters in the same column indicate significant differences between treatments (P< 0.05); **indicates a highly significant effect (P< 0.01), *indicates a significant effect (P< 0.05), and ns indicates no significant effect (P > 0.05); the same applies to **Table 5** below.

### 3.4 Quality indicators

The results of the analysis of the indicators of wolfberry quality in 2021 and 2022 are presented in **Table 5**. In both years, the polysaccharide content in the I_2_N_2_ treatment was 20.97% and 28.05% higher than that in CK. In both years, the total sugar content varied. The highest values occurred in the I_3_N_1_ and I_2_N_1_ treatments, with 10.69% and 9.54% higher values than that in CK. In both years, the I_3_N_1_ treatment had the highest betaine content, with increases of 91.67% and 62.5% relative to that in CK. The I_2_N_2_ treatment had the highest crude fat content, which was 18.67% and 55.36% higher than that in CK. The protein content was the highest in both I_3_N_2_ treatments (2021 and 2022), with an increase of 8.18% and 15.01%, respectively, compared to that in CK.

**Table 5:** The effect of water and nitrogen coupling on quality indicators.

Year	Treatment	Polysaccharide (g·100g^-1^)	Total sugar (g·100g^-1^)	Betaine (g·100g^-1^)	Crude fat (g·100g^-1^)	Protein (g·100g^-1^)
2021	I_1_N_1_	4.61 d	50.43 ab	0.66 bc	1.60 cd	11.00 abc
	I_1_N_2_	4.85 c	50.23 ab	0.76 b	1.70 abc	10.37 cd
	I_1_N_3_	4.79 c	49.10 bc	0.73 b	1.43 cd	10.83 abc
	I_2_N_1_	4.82 c	50.67 ab	0.52 d	1.33 d	10.53 bcd
	I_2_N_2_	5.25 a	49.80 bc	0.57 cd	1.97 a	10.97 abc
	I_2_N_3_	5.07 b	48.70 bc	0.59 cd	1.67 bc	11.27 ab
	I_3_N_1_	5.13 ab	52.61 a	0.92 a	1.70 abc	11.40 a
	I_3_N_2_	5.23 a	50.73 ab	0.67 bc	1.67 bc	11.50 a
	I_3_N_3_	5.17 ab	50.23 ab	0.67 bc	1.93 ab	9.96 d
	CK	4.34 e	47.53 c	0.48 d	1.66 cd	10.63 bcd
F value of ANOVA	I	**	*	**	*	ns
	N	**	*	ns	*	ns
	I×N	*	ns	**	**	**
2022	I_1_N_1_	4.21 de	36.47 bc	0.77 cde	1.14 bcd	10.92 bc
	I_1_N_2_	4.57 c	36.29 bc	0.90 bcd	1.28 bc	9.36 d
	I_1_N_3_	4.41 cd	35.93 bc	0.85 cde	0.96 de	10.75 bc
	I_2_N_1_	4.60 c	38.00 a	0.76 de	0.91 1e	10.63 bc
	I_2_N_2_	5.25 a	36.15 bc	0.78 cde	1.74 a	10.83 bc
	I_2_N_3_	4.82 b	35.44 cd	0.81 cde	1.25 bc	11.00 abc
	I_3_N_1_	5.16 a	37.67 a	1.17 a	1.34 b	11.72 ab
	I_3_N_2_	5.23 a	36.96 ab	1.01 b	1.21 bc	12.03 a
	I_3_N_3_	4.16 e	36.05 bc	0.92 bc	1.21 bc	10.36 cd
	CK	4.10 e	34.69 d	0.72 e	1.12 cd	10.46 c
F value of ANOVA	I	**	ns	**	*	**
	N	**	**	ns	**	ns
	I×N	**	ns	**	**	**

Different lowercase letters in the same column indicate significant differences between the treatments (p < 0.05). ** indicates a highly significant effect (p < 0.01); * indicates a significant effect (p < 0.05); and ns indicates no significant effect (p > 0.05). CK, control; I, irrigation quota; N, amount of nitrogen applied.

### 3.5 Correlation of physiological, growth, quality, and yield indicators

**Figure 9:**
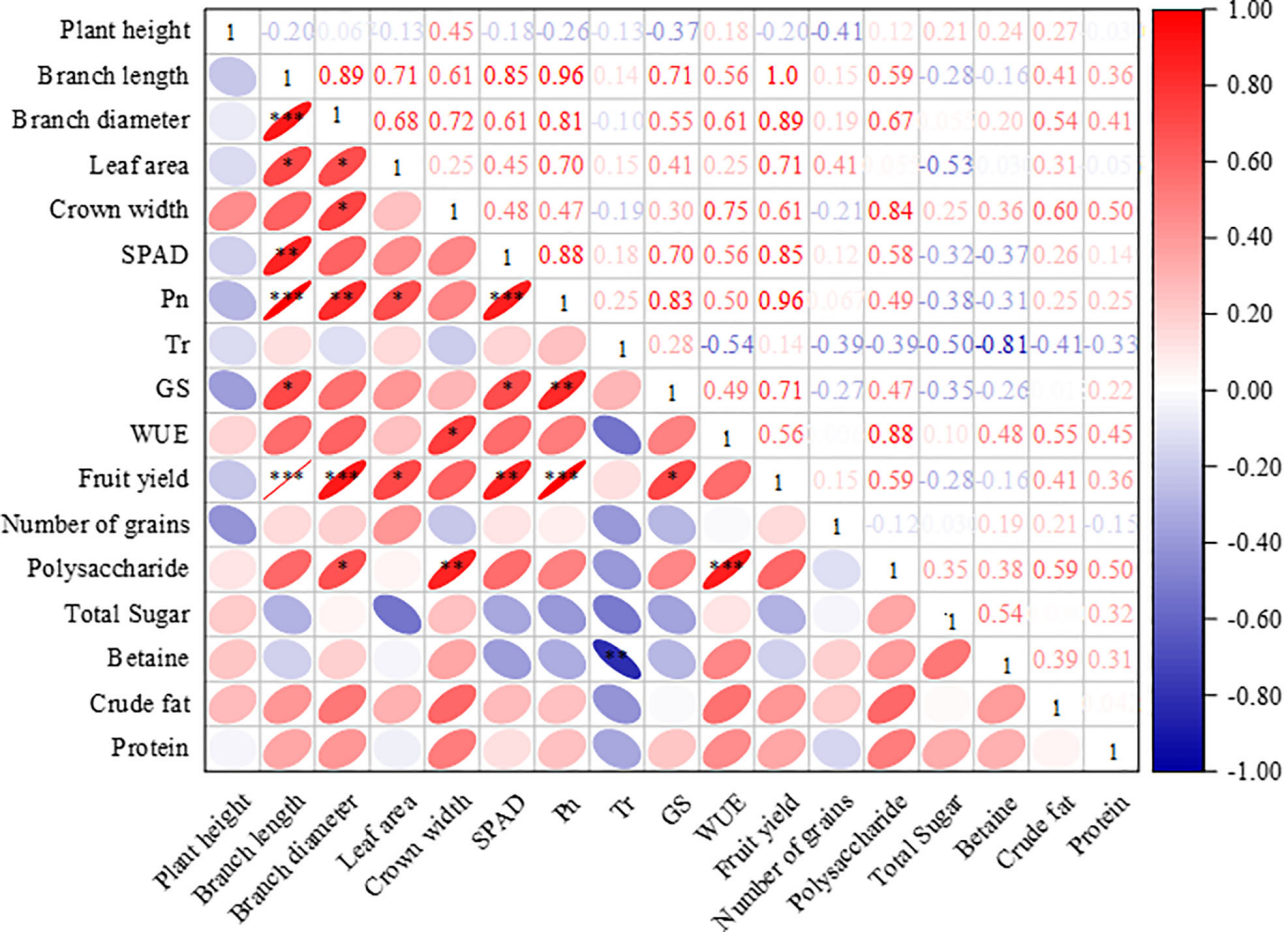
The correlation of physiological, growth, yield, and quality indicators. Note: The data of each indicator is the average of 2021 and 2022; ***indicates highly significant correlation (P< 0.001), **indicates significant correlation (P< 0.01), and *indicates correlation (P< 0.05); *Pn*, Net photosynthetic rate; *Tr*, Transpiration rate; *Gs*, Stomatal conductance and *WUE*, leaf water use efficiency.

To further examine the interactions between the physiology, growth, quality, and yield of wolfberry, we performed correlation analysis with the indices (**Figure 9**). With coefficients of 0.89, 0.96, 1.0, and 0.85, the positive correlations between branch length and branch diameter, *Pn*, yield, and SPAD were highly significant or significant. The branch diameter was positively correlated with polysaccharide, yield, *Pn*, crown width, and leaf area. Significant positive correlations or correlation levels between canopy width, polysaccharides, and *WUE* were observed. With positive correlation coefficients of 0.85 and 0.88, respectively, SPAD showed significant or highly significant correlations with yield and *Pn*. *Pn* demonstrated highly significant or significant positive correlations with yield and *Gs*, as determined by the correlation coefficients of 0.96 and 0.83, respectively. The correlation between *Pn* and betaine was significantly negative, with a correlation coefficient of –0.81.

### 3.6 Comprehensive evaluation

#### 3.6.1 Comprehensive quality evaluation based on the TOPSIS method

All quality indicators were standardized and evaluated based on the TOPSIS method. The overall quality of the treatment was better with a higher optimal treatment *Ci* value. The best overall quality of both years was found in I_3_N_1_, with *Ci* values of 0.821 and 0.682, respectively, except for the I_2_N_1_ treatment, where the overall quality of all other treatments was better than the local conventional management level (CK) (**Table 6**).

**Table 6:** Comprehensive quality evaluation and ranking of wolfberries based on the TOPSIS method.

Year	Treatment	Comprehensive quality indicator					*D_i_^+^*	*D_i_^-^*	*C_i_*	Rank
		Polysaccharide	Total Sugar	Betaine	Crude fat	Protein				
2021	I_1_N_1_	0.296	0.319	0.313	0.303	0.320	0.147	0.108	0.423	7
	I_1_N_2_	0.311	0.318	0.361	0.322	0.302	0.100	0.156	0.609	2
	I_1_N_3_	0.307	0.310	0.347	0.272	0.316	0.140	0.128	0.476	5
	I_2_N_1_	0.309	0.320	0.244	0.253	0.307	0.229	0.044	0.160	10
	I_2_N_2_	0.336	0.315	0.271	0.373	0.319	0.165	0.144	0.466	6
	I_2_N_3_	0.325	0.308	0.279	0.317	0.328	0.168	0.102	0.379	8
	I_3_N_1_	0.329	0.333	0.435	0.323	0.332	0.051	0.231	0.821	1
	I_3_N_2_	0.335	0.321	0.318	0.316	0.335	0.130	0.134	0.507	4
	I_3_N_3_	0.331	0.318	0.315	0.367	0.290	0.129	0.154	0.545	3
	CK	0.278	0.301	0.227	0.297	0.310	0.232	0.048	0.172	9
	Z^+^	0.336	0.333	0.435	0.373	0.335				
	Z^-^	0.278	0.301	0.227	0.253	0.290				
2022	I_1_N_1_	0.285	0.317	0.277	0.292	0.319	0.224	0.079	0.261	7
	I_1_N_2_	0.310	0.315	0.324	0.327	0.273	0.178	0.120	0.404	4
	I_1_N_3_	0.298	0.312	0.305	0.246	0.314	0.241	0.067	0.218	8
	I_2_N_1_	0.312	0.330	0.272	0.233	0.310	0.266	0.059	0.183	10
	I_2_N_2_	0.356	0.314	0.283	0.446	0.316	0.142	0.233	0.620	2
	I_2_N_3_	0.326	0.308	0.293	0.320	0.321	0.185	0.116	0.387	5
	I_3_N_1_	0.349	0.327	0.420	0.343	0.342	0.103	0.221	0.682	1
	I_3_N_2_	0.354	0.321	0.363	0.310	0.351	0.147	0.171	0.538	3
	I_3_N_3_	0.282	0.313	0.332	0.310	0.302	0.185	0.112	0.376	6
	CK	0.278	0.302	0.258	0.285	0.305	0.246	0.062	0.200	9
	Z^+^	0.356	0.330	0.420	0.446	0.351				
	Z^-^	0.278	0.302	0.258	0.233	0.273				

C_i_ indicates the fit degree, Z^+^ indicates the ideal solution, Z^–^ indicates the inverse ideal solution, D_i_^+^ indicates the distance between each treatment and the ideal solution, and D_i_^–^ indicates the distance between each treatment and the inverse ideal solution.

#### 3.6.2 A comprehensive evaluation of the effects of water and nitrogen coupling on the growth, physiology, yield, and quality

Based on the results of the correlation analysis of the branch length, branch diameter, SPAD value, net photosynthetic rate (*Pn*), water use efficiency (*WUE*), yield, and quality, 11 indicators that were highly correlated with the yield and quality of wolfberry were selected. The exhaustive assessment of each treatment was applied to the exhaustive scoring method. Using the coefficient of variation method, the weight distribution of each indicator was determined. Each indicator was assigned a dimensionless individual score to calculate the affiliation, and then, the affiliation of each indicator was multiplied by the weight occupied to determine the overall score. The greater the *C_si_* score, the more effective the treatment. In 2021, the I_2_N_2_ treatment received the highest score (0.81), followed by the I_3_N_2_ treatment (0.78). In 2022, both treatments had the highest and second-highest scores. Based on the two-year average and water conservation goals, the I_2_N_2_ treatment was better (**Table 7**).

**Table 7:** A comprehensive evaluation of the physiology, growth, yield, and quality of wolfberry based on the integrated scoring method.

Year	Treatment	Membership												Comprehensive score *C_si_*	Rank
		Growth indicators		Physiological indicators			Yield	Quality indicators							
		Branch length	Branch diameter	SPAD	*Pn*	WUE	Yield	Polysaccharide	Total Sugar	Betaine	Crude fat	Protein			
2021	I_1_N_1_	0.00	0.00	0.00	0.00	0.00	0.00	0.30	0.57	0.42	0.42	0.68	0.21		10
	I_1_N_2_	0.12	0.24	0.44	0.70	0.70	0.01	0.56	0.53	0.64	0.58	0.26	0.42		6
	I_1_N_3_	0.15	0.59	0.19	0.56	0.28	0.09	0.50	0.31	0.58	0.16	0.57	0.34		8
	I_2_N_1_	0.34	0.55	0.34	0.67	0.27	0.18	0.52	0.62	0.08	0.00	0.37	0.33		9
	I_2_N_2_	1.00	1.00	1.00	1.00	0.76	1.00	1.00	0.45	0.21	1.00	0.65	0.81		1
	I_2_N_3_	0.83	0.80	0.79	0.77	0.22	0.75	0.80	0.23	0.25	0.54	0.85	0.61		4
	I_3_N_1_	0.71	0.77	0.40	0.53	0.52	0.60	0.87	1.00	1.00	0.58	0.94	0.72		3
	I_3_N_2_	0.85	0.97	0.79	0.80	1.00	0.73	0.98	0.63	0.44	0.53	1.00	0.78		2
	I_3_N_3_	0.62	0.81	0.35	0.70	0.69	0.53	0.91	0.53	0.42	0.95	0.00	0.58		5
	CK	0.74	0.75	0.53	0.77	0.40	0.56	0.00	0.00	0.00	0.37	0.44	0.41		7
2022	I_1_N_1_	0.00	0.00	0.00	0.00	0.33	0.00	0.09	0.54	0.12	0.28	0.58	0.17		10
	I_1_N_2_	0.17	0.44	0.39	0.08	0.47	0.12	0.41	0.48	0.41	0.44	0.00	0.33		9
	I_1_N_3_	0.29	0.25	0.26	0.71	0.47	0.24	0.27	0.37	0.29	0.06	0.52	0.33		8
	I_2_N_1_	0.65	0.59	0.52	0.65	0.33	0.43	0.44	1.00	0.08	0.00	0.47	0.44		6
	I_2_N_2_	1.00	0.97	1.00	1.00	0.72	1.00	1.00	0.44	0.15	1.00	0.55	0.78		2
	I_2_N_3_	0.88	0.82	0.82	0.80	1.00	0.82	0.62	0.23	0.21	0.41	0.61	0.63		4
	I_3_N_1_	0.78	0.91	0.40	0.45	0.37	0.57	0.92	0.90	1.00	0.52	0.88	0.71		3
	I_3_N_2_	0.96	1.00	0.81	0.81	0.82	0.88	0.98	0.69	0.65	0.36	1.00	0.80		1
	I_3_N_3_	0.92	0.90	0.45	0.66	0.28	0.65	0.05	0.41	0.46	0.36	0.37	0.48		5
	CK	0.87	0.70	0.67	0.79	0.00	0.82	0.00	0.00	0.00	0.25	0.41	0.37		7

## 4 Discussion

### 4.1 Effect of water and nitrogen coupling on the growth index of wolfberry

Water and nitrogen are extremely important factors affecting crop growth. The proper regulation of water and nitrogen increases water and fertilizer use efficiency and promotes crop growth (Wang et al., 2017; Dai et al., 2019). Plant height, crown width, and branch length are key indicators of the nutritional status of plants and important predictors of fruit yield. In this study, we found that the height, branch diameter, and crown width of wolfberry increased gradually as irrigation water increased. Each indicator continued to increase substantially after the fruiting stage (Jia et al., 2022). In 2021 and 2022, the increase in wolfberry indicators, such as plant height, branch length, and branch diameter, occurred primarily during the spring tip and flowering periods. This increase almost stopped after the fruit-ripening period. The branch length and branch diameter of wolfberry increased as nitrogen application increased in the I_1_ treatment but decreased as nitrogen application increased in the I_2_ and I_3_ treatments. The height of the plants did not change significantly as irrigation and nitrogen application increased. This discrepancy between the findings of our study and those of other studies occurred because, during the pre-growth phase, plant nutrients were primarily allocated to nutritional growth, and as fruit ripening began, competition for water and nutrients developed between reproductive and nutritional growth, resulting in a slowing or cessation of plant growth (Peng et al., 2019). When water is insufficient, a moderate increase in nitrogen can reduce the inhibition of nutrient uptake by water and promote plant growth (Li et al., 2019b). Excessive application of nitrogen in the absence of water stress can reduces the inter-root soil solute potential of the crop to a certain extent, resulting in lower water potential, preventing water and nutrient transport and affecting the nutrient uptake of the crop, while a certain amount of nitrogen application will improve the drought resistance of the crop and thus promote the nutrient uptake of the plant (Wang et al., 2021; Hong et al., 2022). Wolfberry is a perennial plant that can easily adapt. A moderate reduction in the water content did not significantly affect plant height. In contrast, excessive irrigation inhibited the increase in plant height. However, an adequate amount of fertilizer stimulated the growth and development of plant roots (Yin et al., 2018; Landl et al., 2019).

### 4.2 Effect of water and nitrogen coupling on the physiological characteristics of wolfberry

Chlorophyll (SPAD) is necessary for photosynthesis in plants, which is the most efficient process for fixing light energy and is primarily influenced by soil microclimate changes (Sun et al., 2014; Guo et al., 2017; Fan et al., 2019; Gyimah et al., 2020). Studies have shown that increased irrigation and nitrogen application can elevate leaf nitrogen content, and hence leaf chlorophyll content (Zhu et al., 2022). In this study, the SPAD values of wolfberry leaves showed a trend of increasing and then decreasing throughout the reproductive period, and the N_2_ treatment was higher under different irrigation amounts. This is different from the related studies, because of the differences in the nutrient distribution among the organs of the plant at different growth periods, with the nutrients mainly allocated to plant growth and development in the spring tip period and the early flowering period, and the nutrients mainly allocated to fruit growth and development after fruit deposition in the late flowering period, resulting in significant changes in chlorophyll in different growth periods (Zhang, 2021). In addition, wolfberry is a perennial crop; the effect of moderate nitrogen and water reduction on leaf SPAD value is not obvious. The daily dynamics of *Tr* and *Pn* were bimodal with peaks at 10:00 am and noon and 2:00 pm and 4:00 pm, respectively, and the photosynthetic rate performed better under medium to high moisture treatments (Sun et al., 2019; Li et al., 2021a). In this study, *Tr*, *Pn* and *Gs* were unimodal in different fertility periods, all peaking around 10:00 am and noon, and only the *Tr* part of the treatment peaked at 2:00 pm and 4:00 pm. The photosynthetic indices tended to increase and then decrease with the increase of irrigation and nitrogen application, but there were some differences in different fertility periods. The temperature in the study area was already at a high level in all reproductive periods of wolfberry, and the relatively low temperature from 8:00 am and noon, with high stomatal opening of the plants, favored the respiration of the plants and the uptake of water and nutrient elements by the roots, therefore the indices such as *Tr*, *Pn*, and *Gs* gradually increased (Zhu et al., 2022). The plants experience a brief siesta phenomenon, and the photosynthetic daily dynamics of each index gradually decreases when the solar altitude angle reaches its maximum at noon. The solar altitude angle then gradually decreases, but the ground is still a cumulative temperature process and the temperature remains high, coupled with the long age of the plant, the root water supply is not timely, and the leaf water loss is not replenished in time, resulting in a lower frequency of leaf photosynthesis, so it is difficult to have a second peak (Li et al., 2021b).

### 4.3 Effect of water and nitrogen coupling on the yield and quality of wolfberry and comprehensive evaluation

Improving crop yield and quality is the objective of crop cultivation. Several studies have investigated ways to save water and fertilizer while increasing crop yield and quality (Bencze et al., 2014; Sui et al., 2018). Proper irrigation and nitrogen application can increase grain yield and quality. However, the relationship between crop yield, water, and nitrogen is parabolic, implying that water and fertilizer levels above a certain threshold inhibit crop growth and yield (Sandhu et al., 2019; Liu et al., 2020; Liu et al., 2021b). In this study, all yield indicators for 2021 and 2022 first increased and then decreased as irrigation and nitrogen application increased, as found in previous studies. Among the indicators of quality, only the polysaccharide content increased significantly, adding water and nitrogen, whereas the content of the other quality indicators did not increase significantly, which was different from the findings of other studies. Wolfberry is a perennial species, and it can adapt to the arid growing environment as it possesses a good drought-adaptive mechanism. Thus, a moderate reduction in nitrogen and water does not affect its quality (Wang et al., 2018a; Li et al., 2019c). Therefore, quality indicators might be more influenced by soil micronutrients, the appropriate regulation of water and fertilizer, and a favorable growth environment that promotes healthy plant growth and development, which increases crop yield and quality (Wang et al., 2018b; Moreno et al., 2019; Song et al., 2020; Xiao et al., 2021). The I_2_N_2_ treatment enhanced the dry fruit yield and quality indices of polysaccharide and crude fat content of wolfberry to varying degrees. The TOPSIS evaluation showed that the I_3_N_1_ treatment had the highest overall wolfberry quality, but when combined with the scores for growth, physiology, yield, quality, and other indices, as well as the objectives to save water, the I_2_N_2_ treatment was the most effective water and nitrogen management strategy for wolfberry cultivation.

We comprehensively investigated the growth, physiology, yield, and quality of wolfberries in this study. We determined the optimal irrigation quota and nitrogen application for drip irrigation of wolfberry under the coupled regulation of water and nitrogen. However, a comprehensive water and fertilizer management strategy for the wolfberry production system in arid regions still requires optimization. Additionally, wolfberry is extremely sensitive to its growth environment. Thus, the relationship between yield, quality, water and fertilizer utilization efficiency, and the health of the soil needs to be determined to improve the economic efficiency and perform green and efficient development of wolfberry production.

## 5 Conclusion

The increase in the growth indices of wolfberry occurred primarily during the spring tip period and flowering period, and the increase ceased when the fruit ripening period began. Irrigation had the greatest effect, followed by the interaction of water and nitrogen and then nitrogen application, and a moderate increase in irrigation was beneficial to the growth and development of wolfberry plants. Except for the spring tip period, the SPAD value of wolfberry leaves was significantly affected by irrigation and nitrogen application, but the water and nitrogen interaction effect was not significant. The daily dynamics of leaf photosynthetic indicators showed a peak at 10:00 am and noon and a second peak between 2:00 pm and 4:00 pm that was not significant, and the effect of irrigation and nitrogen application during fruit ripening was more significant. Except for the number of grains and total sugar content, all other yield and quality indicators of wolfberry were significantly influenced by irrigation, nitrogen application, and the interaction effect, where the yield of I_2_N_2_ treatment increased by 7.48% and 3.73%, respectively, compared to CK over the two years, and other quality indicators also increased to different degrees. The comprehensive scoring of growth, physiology, yield, and quality indicators, and the water-saving objectives, the irrigation quota, and the nitrogen application rate for multi-objective comprehensive optimization of drip-irrigated wolfberry were found to be 2,565 m^3^ ha^-1^ and 225 kg ha^-1^, which corresponded to the conditions of the I_2_N_2_ treatment. Our findings might provide valuable insights into water and fertilizer management and help in improving the irrigation system of drip-irrigated wolfberries in arid regions.

## Data availability statement

The original contributions presented in the study are included in the article/**Supplementary Material**. Further inquiries can be directed to the corresponding author.

## Author contributions

ZM was mainly responsible for study data collection, analysis, and writing. JY (the corresponding authors) had the overall responsibility for experimental design and project management and overall review of manuscripts. YY, FS, and ZY contributed to data and manuscript preparation. All authors contributed to the article and approved the submitted version.
