# Supplementary material for: Effect of water and nitrogen coupling regulation on the growth, physiology, yield, and quality attributes and comprehensive evaluation of wolfberry (Lycium barbarum L.)
Source: Front Plant Sci. 2023 Jun 21;14:1130109. doi: 10.3389/fpls.2023.1130109 (PMC10320590; doi:10.3389/fpls.2023.1130109)
Supplement: Supplementary file 1 [file DataSheet_1.docx]

Supplementary Material

# Supplementary Data

The original data involved in the article are uploaded in the form of attachments

# Supplementary Figures and Tables

## Supplementary and Tables

Table 1 Physicochemical properties of soils in the study area

| Year | Depth | pH | EC |  | Total N | Total P | Total K | Organic material |  | Ammonium N | Nitrate N |
| --- | --- | --- | --- | --- | --- | --- | --- | --- | --- | --- | --- |
|  | cm |  | μs·cm^-1^ |  | g·kg^-1^ | | | |  | mg·kg^-1^ | |
| 2021 | 0-20 | 8.00 | 1615 |  | 0.46 | 0.43 | 16.19 | 7.58 |  | 12.67 | 14.69 |
|  | 20-40 | 8.20 | 1091 |  | 0.42 | 0.42 | 17.15 | 6.73 |  | 4.51 | 4.73 |
| 2022 | 0-20 | 8.06 | 1524 |  | 0.45 | 0.40 | 17.00 | 7.21 |  | 12.24 | 14.17 |
|  | 20-40 | 8.12 | 963 |  | 0.41 | 0.41 | 17.00 | 6.28 |  | 4.42 | 4.82 |

Table 2. Water and nitrogen management program of Wolfberry

Different treatment

Crop Fertility(date)

|  | | | Irrigation amount mm·ha^-1^ | | | Irrigation ratio % | Nitrogen amount kg·hm^-2^ | | | Nitrogen ratio % |
| --- | --- | --- | --- | --- | --- | --- | --- | --- | --- | --- |
|  |  |  | W1 | W2 | W3 |  | N1 | N2 | N3 |  |
| Spring tip period | 2021 | 04-30 | 43.18 | 51.27 | 59.37 | 20 | 24.75 | 33.75 | 42.75 | 15 |
|  | 2022 | 04-28 | 43.18 | 51.27 | 59.37 | 20 | 24.75 | 33.75 | 42.75 | 15 |
| Flowering period | 2021 | 05-23 | 21.59 | 25.64 | 29.69 | 30 | 12.38 | 16.88 | 21.38 | 25 |
|  |  | 06-18 | 43.18 | 51.27 | 59.37 |  | 28.88 | 39.38 | 49.88 |  |
|  | 2022 | 05-23 | 21.59 | 25.64 | 29.69 | 30 | 12.38 | 16.88 | 21.38 | 25 |
|  |  | 06-15 | 43.18 | 51.27 | 59.37 |  | 28.88 | 39.38 | 49.88 |  |
| Fruit ripening period | 2021 | 07-05 | 21.59 | 25.64 | 29.69 | 40 | 20.63 | 28.13 | 35.63 | 50 |
|  |  | 07-15 | 21.59 | 25.64 | 29.69 |  | 20.63 | 28.13 | 35.63 |  |
|  |  | 07-25 | 21.59 | 25.64 | 29.69 |  | 20.63 | 28.13 | 35.63 |  |
|  |  | 08-05 | 21.59 | 25.64 | 29.69 |  | 20.63 | 28.13 | 35.63 |  |
|  | 2022 | 07-02 | 21.59 | 25.64 | 29.69 | 40 | 20.63 | 28.13 | 35.63 | 50 |
|  |  | 07-15 | 21.59 | 25.64 | 29.69 |  | 20.63 | 28.13 | 35.63 |  |
|  |  | 07-28 | 21.59 | 25.64 | 29.69 |  | 20.63 | 28.13 | 35.63 |  |
|  |  | 08-08 | 21.59 | 25.64 | 29.69 |  | 20.63 | 28.13 | 35.63 |  |
| Defoliation period | 2021 | 08-25 | 21.59 | 25.64 | 29.69 | 10 | 16.50 | 22.50 | 28.50 | 10 |
|  | 2022 | 08-28 | 21.59 | 25.64 | 29.69 | 10 | 16.50 | 22.50 | 28.50 |  |
| Total | 2021 | | 215.89 | 256.37 | 296.85 | 100 | 165 | 225 | 285 | 100 |
|  | 2022 | |  |  |  | 100 |  |  |  | 100 |

Table 3. Analysis of variance for growth indicators under water and nitrogen coupling

| Indices | Treatment | Spring tip period | |  | Flowering period | |  | Fruit ripening period | |
| --- | --- | --- | --- | --- | --- | --- | --- | --- | --- |
|  |  | 2021 | 2022 |  | 2021 | 2022 |  | 2021 | 2022 |
| Plant height | W | 4.97** | 17.57** |  | 7.31** | 3.30* |  | 0.26ns | 22.39** |
|  | N | 2.29ns | 3.28* |  | 3.23* | 13.3** |  | 1.23ns | 0.13ns |
|  | W×N | 6.45** | 2.75* |  | 1.05ns | 2.19ns |  | 2.36ns | 16.05** |
| Crown Width (East - West) | W | 11.59** | 18.17** |  | 4.43* | 0.57ns |  | 12.18** | 6.22** |
|  | N | 0.12ns | 15.64** |  | 0.09ns | 3.95* |  | 10.74** | 0.31ns |
|  | W×N | 2.62* | 10.58** |  | 1.91ns | 11.30** |  | 6.09** | 9.48** |
| Crown Width (South - North) | W | 9.35** | 14.96** |  | 0.45ns | 2.86ns |  | 0.84ns | 1.37ns |
|  | N | 0.47ns | 1.03ns |  | 0.19ns | 1.82ns |  | 0.12ns | 0.18ns |
|  | W×N | 5.12** | 2.75* |  | 1.94ns | 0.35ns |  | 0.84ns | 0.40ns |
| Branch length | W | 14.05** | 32.35** |  | 16.68** | 14.14** |  | 6.92** | 6.01** |
|  | N | 2.92ns | 1.10ns |  | 6.48** | 3.17* |  | 2.15ns | 11.16** |
|  | W×N | 1.02ns | 1.83ns |  | 3.78** | 0.87ns |  | 2.92* | 0.23ns |
| Branch diameter | W | 2.92ns | 5.40** |  | 6.31** | 11.32** |  | 0.66ns | 0.02ns |
|  | N | 2.48ns | 2.87ns |  | 1.06ns | 1.01ns |  | 0.51ns | 0.88ns |
|  | W×N | 0.04ns | 1.03ns |  | 0.46ns | 0.53ns |  | 0.47ns | 0.41ns |

Note: ** indicates a highly significant level (P<0.01), * indicates a significant level (P<0.05), ns indicates no significant effect (P>0.05); W, the irrigation quota; N, the amount of nitrogen applied; the same below.

Table 4 Effect of water and nitrogen coupling on yield and yield composition

| Year | Treatment | Fruit yield (kg·hm^-2^) | Hundred Grains Weight (g) | Dry to fresh ratio | Number of grains (50g) |
| --- | --- | --- | --- | --- | --- |
| 2021 | W1N1 | 1953.00 ± 18.19c | 17.40 ± 0.35abc | 4.26 ± 0.03b | 293.00 ± 5.00abc |
|  | W1N2 | 1956.16 ± 31.56c | 16.79 ± 0.40c | 4.28 ± 0.03ab | 295.67 ± 9.71ab |
|  | W1N3 | 1984.41 ± 59.26c | 18.21 ± 0.24a | 4.32 ± 0.03a | 279.67 ± 3.79c |
|  | W2N1 | 2020.84 ± 24.83c | 17.91 ± 0.41ab | 4.29 ± 0.04ab | 282.00 ± 13.89bc |
|  | W2N2 | 2320.94 ± 83.25a | 17.24 ± 0.12bc | 4.28 ± 0.03ab | 288.67 ± 3.51abc |
|  | W2N3 | 2230.26 ± 30.88ab | 17.59 ± 0.44abc | 4.26 ± 0.04b | 290.67 ± 10.07abc |
|  | W3N1 | 2172.74 ± 21.01b | 17.39 ± 0.29bc | 4.13 ± 0.01d | 293.67 ± 9.02abc |
|  | W3N2 | 2221.63 ± 21.73ab | 17.96 ± 0.82ab | 4.20 ± 0.04c | 297.33 ± 6.03ab |
|  | W3N3 | 2148.89 ± 64.21b | 17.25 ± 0.41bc | 4.25 ± 0.01bc | 297.33 ± 8.02ab |
|  | CK | 2159.34 ± 124.35b | 17.34 ± 0.46bc | 4.32 ± 0.03a | 302.33 ± 8.14a |
| F value of ANOVA | W | 43.50** | 0.17ns | 25.83** | 2.85ns |
|  | N | 9.33** | 1.61ns | 6.47** | 0.89ns |
|  | W×N | 6.58** | 5.40** | 5.12** | 1.70ns |
| 2022 | W1N1 | 1924.87 ± 49.44f | 14.95 ± 0.35c | 4.18 ± 0.04d | 321.33 ± 6.81ab |
|  | W1N2 | 1983.14 ± 43.06ef | 15.01 ± 0.60c | 4.23 ± 0.04bcd | 322.00 ± 9.85ab |
|  | W1N3 | 2035.97 ± 75.56def | 15.34 ± 0.17bc | 4.29 ± 0.06bcd | 310.33 ± 6.66b |
|  | W2N1 | 2125.74 ± 90.10cde | 15.34 ± 0.83bc | 4.27 ± 0.04bcd | 311.67 ± 8.02b |
|  | W2N2 | 2391.73 ± 101.98a | 16.54 ± 0.84a | 4.20 ± 0.05cd | 315.33 ± 4.16b |
|  | W2N3 | 2307.75 ± 55.52ab | 16.34 ± 0.71ab | 4.19 ± 0.05cd | 308.33 ± 14.47b |
|  | W3N1 | 2192.29 ± 74.55bcd | 15.23 ± 0.36c | 4.34 ± 0.03abc | 319.33 ± 6.11ab |
|  | W3N2 | 2333.85 ± 183.40ab | 15.66 ± 0.31abc | 4.36 ± 0.07ab | 323.33 ± 7.09ab |
|  | W3N3 | 2227.90 ± 47.21abc | 15.80 ± 1.35abc | 4.38 ± 0.10ab | 319.33 ± 28.02ab |
|  | CK | 2305.82 ± 75.77ab | 14.77 ± 0.49c | 4.48 ± 0.19a | 338.33 ± 8.14a |
| F value of ANOVA | W | 30.42** | 7.07** | 8.40** | 1.31ns |
|  | N | 7.28** | 3.80* | 0.24ns | 0.93ns |
|  | W×N | 1.5ns | 0.885ns | 1.11ns | 0.18ns |

Note: Different lowercase letters in the same column indicate significant differences between treatments (P < 0.05), ** indicates a highly significant level (P < 0.01), * indicates a significant level (P < 0.05), ns indicates no significant effect (P > 0.05), the same below.

Table 5 Effect of water and nitrogen coupling on quality indicators

| Year | Treatment | Polysaccharide (g·100g^-1^) | Total Sugar (g·100g^-1^) | Betaine (g·100g^-1^) | Crude fat (g·100g^-1^) | Protein (g·100g^-1^) |
| --- | --- | --- | --- | --- | --- | --- |
| 2021 | W1N1 | 4.61 ± 0.02d | 50.43 ± 0.90ab | 0.66 ± 0.04bc | 1.60 ± 0.10cd | 11.00 ± 0.36abc |
|  | W1N2 | 4.85 ± 0.14c | 50.23 ± 1.75ab | 0.76 ± 0.07b | 1.70 ± 0.10abc | 10.37 ± 0.12cd |
|  | W1N3 | 4.79 ± 0.05c | 49.10 ± 0.86bc | 0.73 ± 0.05b | 1.43 ± 0.15cd | 10.83 ± 0.40abc |
|  | W2N1 | 4.82 ± 0.04c | 50.67 ± 0.85ab | 0.52 ± 0.04d | 1.33 ± 0.25d | 10.53 ± 0.15bcd |
|  | W2N2 | 5.25 ± 0.02a | 49.80 ± 1.87bc | 0.57 ± 0.07cd | 1.97 ± 0.15a | 10.97 ± 0.25abc |
|  | W2N3 | 5.07 ± 0.04b | 48.70 ± 1.79bc | 0.59 ± 0.08cd | 1.67 ± 0.08bc | 11.27 ± 0.66ab |
|  | W3N1 | 5.13 ± 0.02ab | 52.61 ± 0.99a | 0.92 ± 0.08a | 1.70 ± 0.15abc | 11.40 ± 0.26a |
|  | W3N2 | 5.23 ± 0.16a | 50.73 ± 0.87ab | 0.67 ± 0.12bc | 1.67 ± 0.06bc | 11.50 ± 0.70a |
|  | W3N3 | 5.17 ± 0.05ab | 50.23 ± 1.01ab | 0.67 ± 0.12bc | 1.93 ± 0.25ab | 9.96 ± 0.33d |
|  | CK | 4.34 ± 0.11e | 47.53 ± 0.91c | 0.48 ± 0.05d | 1.66 ± 0.08cd | 10.63 ± 0.15bcd |
| F value of ANOVA | W | 64.07** | 3.66* | 19.41** | 3.53* | 0.83ns |
|  | N | 22.34** | 5.14* | 0.69ns | 5.27* | 1.49ns |
|  | W×N | 3.51* | 0.36ns | 7.21** | 6.38** | 8.92** |
| 2022 | W1N1 | 4.21 ± 0.11de | 36.47 ± 0.39bc | 0.77 ± 0.05cde | 1.14 ± 0.15bcd | 10.92 ± 0.16bc |
|  | W1N2 | 4.57 ± 0.15c | 36.29 ± 0.71bc | 0.90 ± 0.07bcd | 1.28 ± 0.05bc | 9.36 ± 0.21d |
|  | W1N3 | 4.41 ± 0.12cd | 35.93 ± 0.51bc | 0.85 ± 0.09cde | 0.96 ± 0.09de | 10.75 ± 0.25bc |
|  | W2N1 | 4.60 ± 0.13c | 38.00 ± 0.43a | 0.76 ± 0.14de | 0.91 ± 0.11e | 10.63 ± 0.51bc |
|  | W2N2 | 5.25 ± 0.10a | 36.15 ± 0.50bc | 0.78 ± 0.08cde | 1.74 ± 0.16a | 10.83 ± 0.82bc |
|  | W2N3 | 4.82 ± 0.12b | 35.44 ± 0.80cd | 0.81 ± 0.03cde | 1.25 ± 0.18bc | 11.00 ± 0.64abc |
|  | W3N1 | 5.16 ± 0.11a | 37.67 ± 0.55a | 1.17 ± 0.12a | 1.34 ± 0.10b | 11.72 ± 0.730ab |
|  | W3N2 | 5.23 ± 0.09a | 36.96 ± 0.10ab | 1.01 ± 0.02b | 1.21 ± 0.05bc | 12.03 ± 0.76a |
|  | W3N3 | 4.16 ± 0.10e | 36.05 ± 1.19bc | 0.92 ± 0.08bc | 1.21 ± 0.07bc | 10.36 ± 0.89cd |
|  | CK | 4.10 ± 0.17e | 34.69 ± 0.11d | 0.72 ± 0.15e | 1.12 ± 0.11cd | 10.46 ± 0.51c |
| F value of ANOVA | W | 46.19** | 2.67ns | 23.90** | 5.51* | 6.53** |
|  | N | 48.92** | 15.02** | 0.64ns | 17.00** | 1.12ns |
|  | W×N | 26.78** | 2.47ns | 4.54** | 14.65** | 5.88** |

Table 6 Comprehensive quality evaluation and ranking of Wolfberry under water and nitrogen coupling based on TOPSIS method

| year | Treatment | Comprehensive quality indicator | | | | | *D_i_^+^* | *D_i_^-^* | *C_i_* | Rank |
| --- | --- | --- | --- | --- | --- | --- | --- | --- | --- | --- |
|  |  | Polysaccharide | Total Sugar | Betaine | Crude fat | Protein |  |  |  |  |
| 2021 | W1N1 | 0.296 | 0.319 | 0.313 | 0.303 | 0.320 | 0.147 | 0.108 | 0.423 | 7 |
|  | W1N2 | 0.311 | 0.318 | 0.361 | 0.322 | 0.302 | 0.100 | 0.156 | 0.609 | 2 |
|  | W1N3 | 0.307 | 0.310 | 0.347 | 0.272 | 0.316 | 0.140 | 0.128 | 0.476 | 5 |
|  | W2N1 | 0.309 | 0.320 | 0.244 | 0.253 | 0.307 | 0.229 | 0.044 | 0.160 | 10 |
|  | W2N2 | 0.336 | 0.315 | 0.271 | 0.373 | 0.319 | 0.165 | 0.144 | 0.466 | 6 |
|  | W2N3 | 0.325 | 0.308 | 0.279 | 0.317 | 0.328 | 0.168 | 0.102 | 0.379 | 8 |
|  | W3N1 | 0.329 | 0.333 | 0.435 | 0.323 | 0.332 | 0.051 | 0.231 | 0.821 | 1 |
|  | W3N2 | 0.335 | 0.321 | 0.318 | 0.316 | 0.335 | 0.130 | 0.134 | 0.507 | 4 |
|  | W3N3 | 0.331 | 0.318 | 0.315 | 0.367 | 0.290 | 0.129 | 0.154 | 0.545 | 3 |
|  | CK | 0.278 | 0.301 | 0.227 | 0.297 | 0.310 | 0.232 | 0.048 | 0.172 | 9 |
|  | Z^+^ | 0.336 | 0.333 | 0.435 | 0.373 | 0.335 |  |  |  |  |
|  | Z^-^ | 0.278 | 0.301 | 0.227 | 0.253 | 0.290 |  |  |  |  |
| 2022 | W1N1 | 0.285 | 0.317 | 0.277 | 0.292 | 0.319 | 0.224 | 0.079 | 0.261 | 7 |
|  | W1N2 | 0.310 | 0.315 | 0.324 | 0.327 | 0.273 | 0.178 | 0.120 | 0.404 | 4 |
|  | W1N3 | 0.298 | 0.312 | 0.305 | 0.246 | 0.314 | 0.241 | 0.067 | 0.218 | 8 |
|  | W2N1 | 0.312 | 0.330 | 0.272 | 0.233 | 0.310 | 0.266 | 0.059 | 0.183 | 10 |
|  | W2N2 | 0.356 | 0.314 | 0.283 | 0.446 | 0.316 | 0.142 | 0.233 | 0.620 | 2 |
|  | W2N3 | 0.326 | 0.308 | 0.293 | 0.320 | 0.321 | 0.185 | 0.116 | 0.387 | 5 |
|  | W3N1 | 0.349 | 0.327 | 0.420 | 0.343 | 0.342 | 0.103 | 0.221 | 0.682 | 1 |
|  | W3N2 | 0.354 | 0.321 | 0.363 | 0.310 | 0.351 | 0.147 | 0.171 | 0.538 | 3 |
|  | W3N3 | 0.282 | 0.313 | 0.332 | 0.310 | 0.302 | 0.185 | 0.112 | 0.376 | 6 |
|  | CK | 0.278 | 0.302 | 0.258 | 0.285 | 0.305 | 0.246 | 0.062 | 0.200 | 9 |
|  | Z^+^ | 0.356 | 0.330 | 0.420 | 0.446 | 0.351 |  |  |  |  |
|  | Z^-^ | 0.278 | 0.302 | 0.258 | 0.233 | 0.273 |  |  |  |  |

Note: *C_i_* is the fit degree, Z^＋^ is the ideal solution, Z^-^ is the inverse ideal solution; D_i_^＋^ is the distance between each treatment and the ideal solution, and D_i_^-^ is the distance between each treatment and the inverse ideal solution.

Table 7 Comprehensive evaluation of physiology, growth, yield and quality of Wolfberry based on integrated scoring method

| year | Treatment | Membership | | | | | | | | | | | | | | Comprehensive score *C_si_* | Rank |
| --- | --- | --- | --- | --- | --- | --- | --- | --- | --- | --- | --- | --- | --- | --- | --- | --- | --- |
|  |  | Growth indicators | |  | Physiological indicators | | |  | Yield |  | Quality indicators | | | | |  |  |
|  |  | Branch length | Branch diameter |  | SPAD | Pn | WUE |  | Yield |  | Polysaccharide | Total Sugar | Betaine | Crude fat | Protein |  |  |
| 2021 | W1N1 | 0.00 | 0.00 |  | 0.00 | 0.00 | 0.00 |  | 0.00 |  | 0.30 | 0.57 | 0.42 | 0.42 | 0.68 | 0.21 | 10 |
|  | W1N2 | 0.12 | 0.24 |  | 0.44 | 0.70 | 0.70 |  | 0.01 |  | 0.56 | 0.53 | 0.64 | 0.58 | 0.26 | 0.42 | 6 |
|  | W1N3 | 0.15 | 0.59 |  | 0.19 | 0.56 | 0.28 |  | 0.09 |  | 0.50 | 0.31 | 0.58 | 0.16 | 0.57 | 0.34 | 8 |
|  | W2N1 | 0.34 | 0.55 |  | 0.34 | 0.67 | 0.27 |  | 0.18 |  | 0.52 | 0.62 | 0.08 | 0.00 | 0.37 | 0.33 | 9 |
|  | W2N2 | 1.00 | 1.00 |  | 1.00 | 1.00 | 0.76 |  | 1.00 |  | 1.00 | 0.45 | 0.21 | 1.00 | 0.65 | 0.81 | 1 |
|  | W2N3 | 0.83 | 0.80 |  | 0.79 | 0.77 | 0.22 |  | 0.75 |  | 0.80 | 0.23 | 0.25 | 0.54 | 0.85 | 0.61 | 4 |
|  | W3N1 | 0.71 | 0.77 |  | 0.40 | 0.53 | 0.52 |  | 0.60 |  | 0.87 | 1.00 | 1.00 | 0.58 | 0.94 | 0.72 | 3 |
|  | W3N2 | 0.85 | 0.97 |  | 0.79 | 0.80 | 1.00 |  | 0.73 |  | 0.98 | 0.63 | 0.44 | 0.53 | 1.00 | 0.78 | 2 |
|  | W3N3 | 0.62 | 0.81 |  | 0.35 | 0.70 | 0.69 |  | 0.53 |  | 0.91 | 0.53 | 0.42 | 0.95 | 0.00 | 0.58 | 5 |
|  | CK | 0.74 | 0.75 |  | 0.53 | 0.77 | 0.40 |  | 0.56 |  | 0.00 | 0.00 | 0.00 | 0.37 | 0.44 | 0.41 | 7 |
| 2022 | W1N1 | 0.00 | 0.00 |  | 0.00 | 0.00 | 0.33 |  | 0.00 |  | 0.09 | 0.54 | 0.12 | 0.28 | 0.58 | 0.17 | 10 |
|  | W1N2 | 0.17 | 0.44 |  | 0.39 | 0.08 | 0.47 |  | 0.12 |  | 0.41 | 0.48 | 0.41 | 0.44 | 0.00 | 0.33 | 9 |
|  | W1N3 | 0.29 | 0.25 |  | 0.26 | 0.71 | 0.47 |  | 0.24 |  | 0.27 | 0.37 | 0.29 | 0.06 | 0.52 | 0.33 | 8 |
|  | W2N1 | 0.65 | 0.59 |  | 0.52 | 0.65 | 0.33 |  | 0.43 |  | 0.44 | 1.00 | 0.08 | 0.00 | 0.47 | 0.44 | 6 |
|  | W2N2 | 1.00 | 0.97 |  | 1.00 | 1.00 | 0.72 |  | 1.00 |  | 1.00 | 0.44 | 0.15 | 1.00 | 0.55 | 0.78 | 2 |
|  | W2N3 | 0.88 | 0.82 |  | 0.82 | 0.80 | 1.00 |  | 0.82 |  | 0.62 | 0.23 | 0.21 | 0.41 | 0.61 | 0.63 | 4 |
|  | W3N1 | 0.78 | 0.91 |  | 0.40 | 0.45 | 0.37 |  | 0.57 |  | 0.92 | 0.90 | 1.00 | 0.52 | 0.88 | 0.71 | 3 |
|  | W3N2 | 0.96 | 1.00 |  | 0.81 | 0.81 | 0.82 |  | 0.88 |  | 0.98 | 0.69 | 0.65 | 0.36 | 1.00 | 0.80 | 1 |
|  | W3N3 | 0.92 | 0.90 |  | 0.45 | 0.66 | 0.28 |  | 0.65 |  | 0.05 | 0.41 | 0.46 | 0.36 | 0.37 | 0.48 | 5 |
|  | CK | 0.87 | 0.70 |  | 0.67 | 0.79 | 0.00 |  | 0.82 |  | 0.00 | 0.00 | 0.00 | 0.25 | 0.41 | 0.37 | 7 |

## Supplementary Figures

Fig. 1 Daily rainfall and daily average temperature during the reproductive period of Wolfberry 2021 and 2022


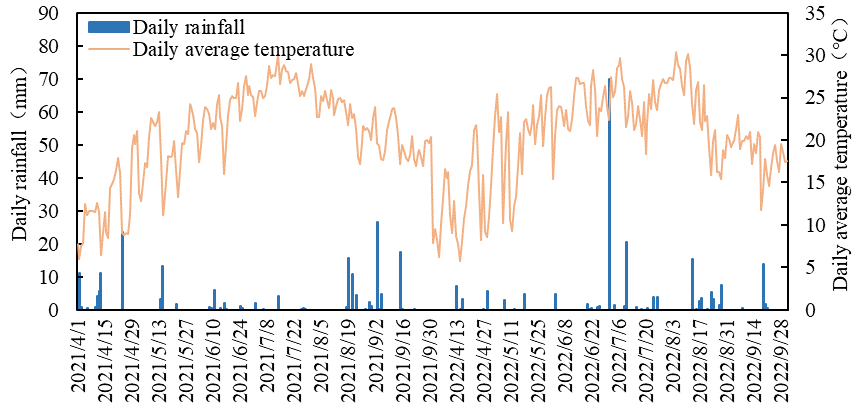

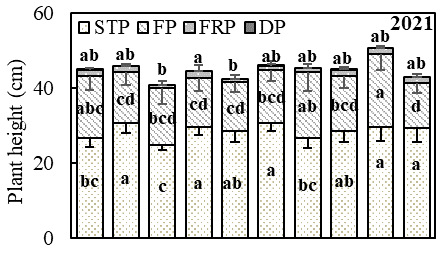

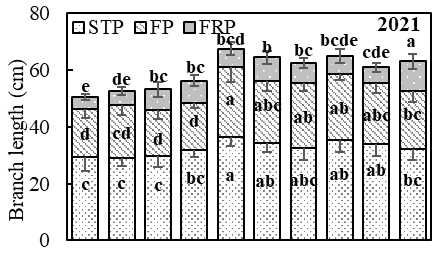

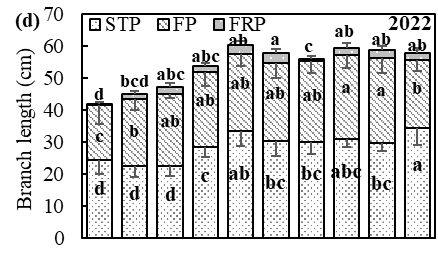

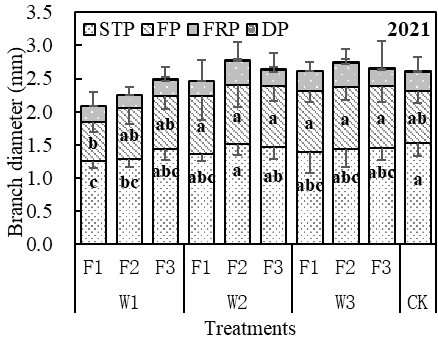

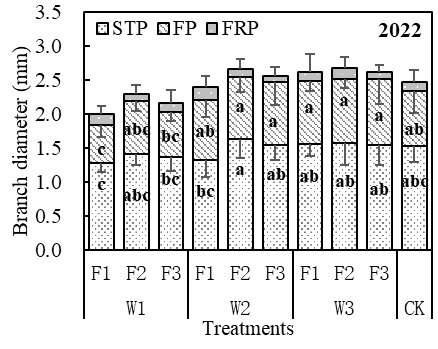


**(a)**

**(c)**

**(f)**

**(e)**


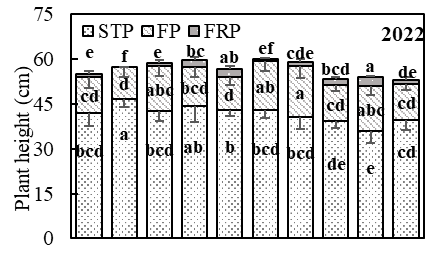


**(b)**

Note: The different lowercase indicate significant differences according to Duncan’s multiple-range test ( P＜0.05).STP, Spring tip period; FP, Flowering period; FRS, Fruit ripening stage; DP, Defoliation period.

Fig. 2 Effect of water and nitrogen interactions on the cumulative growth

Note: ** and * indicate significant differences at the (P < 0.01) and (P < 0.05) levels, respectively.

**a**

**b**

Fig. 3 Effect of water and nitrogen coupling on SPAD value


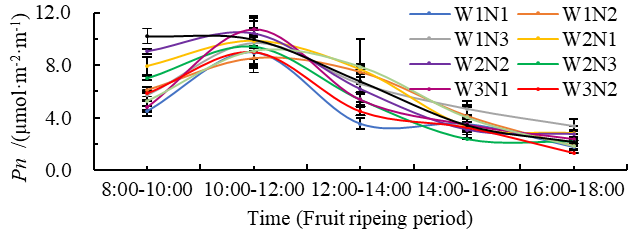

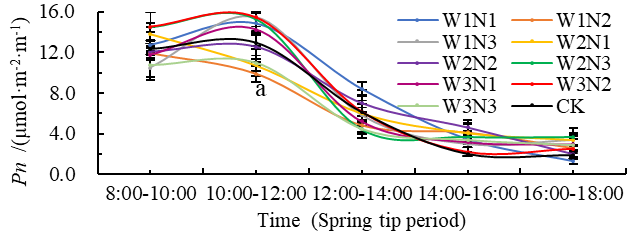

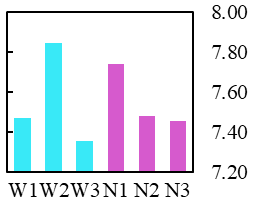

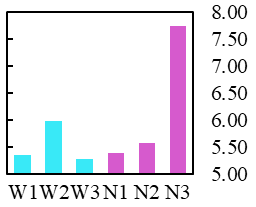


**a**

**b**

Note: Photosynthetic day dynamics at different fertility stages were expressed as average values in 2021 and 2022, below.

Fig. 4 Daily dynamic characteristics and trends of net photosynthetic rate under water and nitrogen coupling


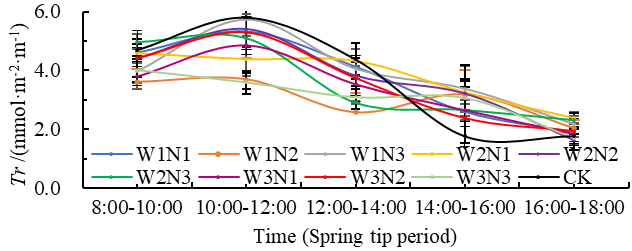

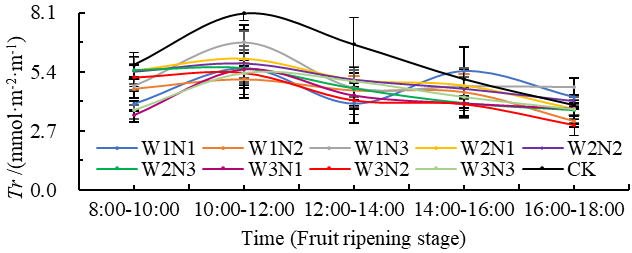

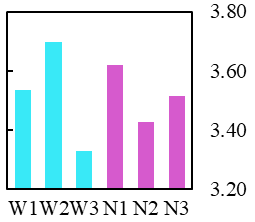

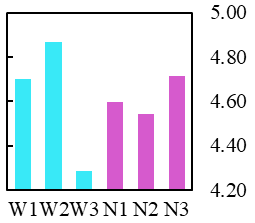


a

b

Fig. 5 Daily dynamic characteristics and trend of transpiration rate under water and nitrogen coupling


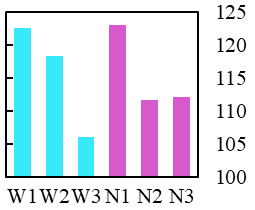

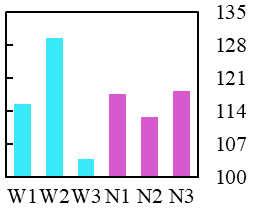

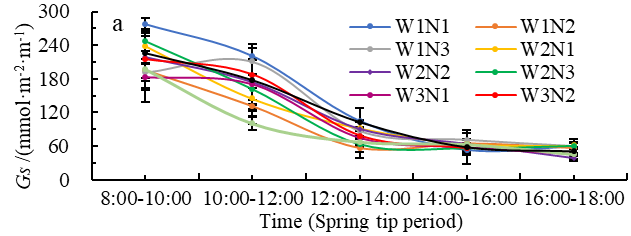

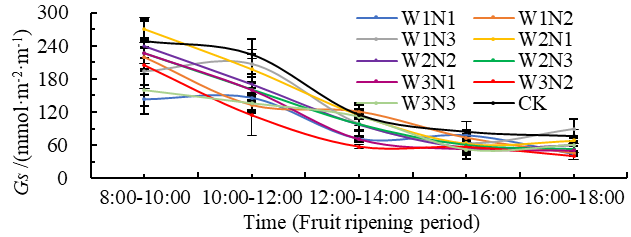


b

Fig. 6 Daily dynamic characteristics and trends of stomatal conductance under water and nitrogen coupling


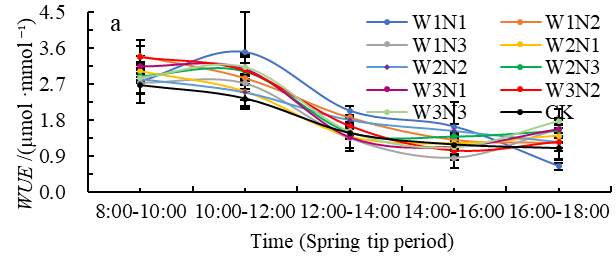

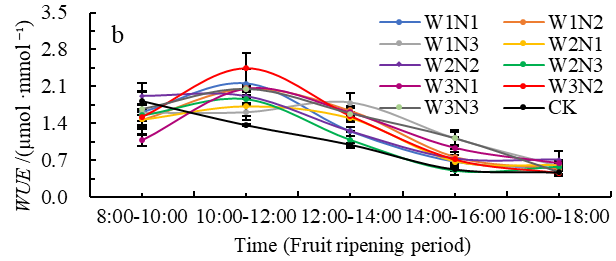

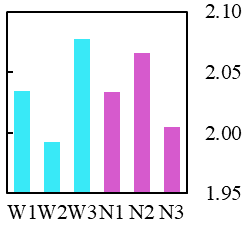

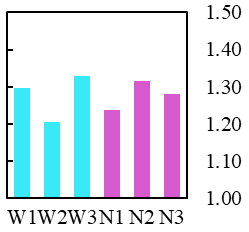


Fig. 7 Daily dynamic characteristics and trends of water use efficiency under water and nitrogen coupling


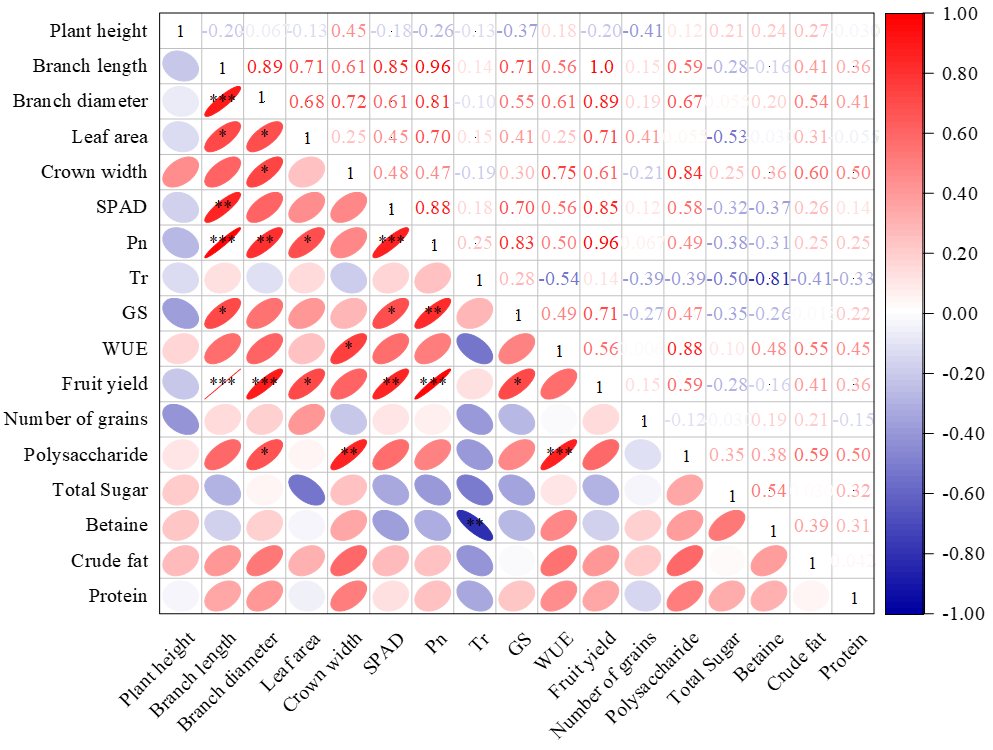
Note: *** indicates highly significant correlation (P < 0.001), ** indicates significant correlation (P < 0.01), * indicates correlation (P < 0.05); data of each indicator is the average of 2021 and 2022.

Fig. 8 Correlation of physiological, growth, yield and quality indicators
